# DNMT3L inhibits hepatocellular carcinoma progression through DNA methylation of CDO1: insights from big data to basic research

**DOI:** 10.1186/s12967-024-04939-9

**Published:** 2024-02-02

**Authors:** Xiaokai Yan, Yao Qi, Xinyue Yao, Nanjing Zhou, Xinxin Ye, Xing Chen

**Affiliations:** 1 Department of Oncology, The Second Affiliated Hospital of Zunyi Medical University, Zunyi, China; 2 Shanghai Molecular Medicine Engineering Technology Research Center, Shanghai, 201203 China; 3 Shanghai National Engineering Research Center of Biochip, Shanghai, 201203 China; 4 Department of Hepatopancreatobiliary Surgery, Zhejiang Cancer Hospital, Hangzhou Institute of Medicine (HIM), Chinese Academy of Sciences, Hangzhou, 310022 Zhejiang China

**Keywords:** Hepatocellular carcinoma, Big data, DNA methylation, DNMT3L, CDO1

## Abstract

**Background:**

DNMT3L is a crucial DNA methylation regulatory factor, yet its function and mechanism in hepatocellular carcinoma (HCC) remain poorly understood. Bioinformatics-based big data analysis has increasingly gained significance in cancer research. Therefore, this study aims to elucidate the role of DNMT3L in HCC by integrating big data analysis with experimental validation.

**Methods:**

Dozens of HCC datasets were collected to analyze the expression of DNMT3L and its relationship with prognostic indicators, and were used for molecular regulatory relationship evaluation. The effects of DNMT3L on the malignant phenotypes of hepatoma cells were confirmed in vitro and in vivo. The regulatory mechanisms of DNMT3L were explored through MSP, western blot, and dual-luciferase assays.

**Results:**

DNMT3L was found to be downregulated in HCC tissues and associated with better prognosis. Overexpression of DNMT3L inhibits cell proliferation and metastasis. Additionally, CDO1 was identified as a target gene of DNMT3L and also exhibits anti-cancer effects. DNMT3L upregulates CDO1 expression by competitively inhibiting DNMT3A-mediated methylation of CDO1 promoter.

**Conclusions:**

Our study revealed the role and epi-transcriptomic regulatory mechanism of DNMT3L in HCC, and underscored the essential role and applicability of big data analysis in elucidating complex biological processes.

**Supplementary Information:**

The online version contains supplementary material available at 10.1186/s12967-024-04939-9.

## Introduction

Hepatocellular carcinoma (HCC) is currently one of the most common malignant tumors worldwide, with incidence and mortality rates ranking sixth and third, respectively, resulting in approximately 906,000 new cases and 830,000 deaths annually [1]. The disease poses a significant threat to the health and lives of people. Treatment options for HCC include hepatectomy, liver transplantation, local radiofrequency ablation, transarterial chemoembolization (TACE), systemic chemotherapy, immunotherapy, and targeted therapy. However, due to the lack of specific early clinical symptoms and signs, early diagnosis of HCC remains challenging, leading to most patients being diagnosed in the middle or late stages with a low 5-year survival rate. In addition, HCC is a hypervascular tumor that is prone to metastasis, which causes patients to lose the opportunity for surgery and leads to a poor prognosis. Thus, investigating the key molecules and their regulatory mechanisms involved in the growth and metastasis process of HCC has crucial clinical implications for improving the survival benefits and life quality of patients.

In the past, cancer research mainly focused on studying a limited number of crucial pathways and genes at a molecular and clinical level. Recent years have seen a rapid accumulation of large-scale cancer omics data, driven by breakthroughs in high-throughput technologies. These advancements have significantly enhanced our understanding of cancer biology and led to translational advancements [2]. High-throughput technologies refer to techniques that can process large amounts of samples or data simultaneously, such as gene chips, high-throughput sequencing, and protein arrays. In life science research, these technologies can be used to study the expression, function, and interactions of molecules such as genes, proteins, and metabolites, thereby revealing the mechanisms of biological phenomena [3, 4]. In 2011, Mínguez et al. [5] identified 35 genes that were significantly associated with vascular invasion in HCC using gene chips. Over the last decade, several of these genes have been discovered to play a role in HCC growth and metastasis [6–9], providing important insights into the underlying mechanisms of HCC development. Indeed, results obtained from high-throughput data analysis are not always reliable. PIK3R1, one of the 35 genes mentioned above, was initially considered a tumor suppressor gene due to its low expression in tumor samples without vascular invasion [5]. However, Ai et al.'s research [10] found that it can actually promote tumor proliferation and invasion, exhibiting oncogenic functions. Typically, the credibility of data analysis results heavily depends on the size of the dataset. Disease research is often limited by technical, ethical, or other factors, and the amount of biological samples collected is usually small, ranging from tens to hundreds. Furthermore, since different studies have variations in backgrounds, experimental techniques, and other aspects, results based on individual or a small number of data points are often not robust. Hence, an increasing number of studies indicate that combining data from multiple centers or studies can achieve more reliable results and reveal new insights, especially when individual datasets are affected by noise, incompleteness, or bias caused by certain artifacts [2]. This is the application of ‘big data’ in life science.

DNA methylation is a widespread epigenetic modification that exists in eukaryotic DNA. It involves the addition of a methyl group to the fifth carbon atom of cytosine residues in CpG dinucleotides, forming 5-methylcytosine (5mC) [11]. This process is catalyzed by DNA methyltransferases (DNMTs). Currently, the DNMTs identified in mammals mainly include DNMT1, DNMT3A, and DNMT3B, among which DNMT1 is mainly involved in maintaining the DNA methylation status [12], while DNMT3A and DNMT3B are mainly involved in de novo DNA methylation [13]. Additionally, the DNA methylation process can be reversed by the DNA demethylase TET family proteins (including TET1, TET2, and TET3) [14]. Numerous studies have demonstrated that DNA methylation plays a crucial role in various biological processes, including gene expression, genomic imprinting, transposon silencing, and maintenance of chromosome structure [15–17]. It is also involved in regulating a diverse range of biological behaviors, such as tissue embryonic development, vascular formation, immune response, tumor initiation, and metastasis [18–21]. DNMT1 can be recruited by EZH2 to the promoter of miR-200b/a/429, mediating methylation silencing and promoting tumor progression [22]. DNMT3B can suppress the expression of IRF8 through DNA methylation, leading to the occurrence of inflammation-related colon cancer [23]. The TET family proteins can suppress breast cancer metastasis by downregulating the methylation of the miR-200 promoter [24]. In summary, DNA methylation plays a critical role in the growth and metastasis of tumors, but the specific molecular mechanisms involved still require further research. DNA methyltransferase 3-like (DNMT3L) is an important regulatory factor in the methylation process, and the prevailing view is that it can enhance DNMT3A activity to promote DNA methylation [25, 26]. Previous studies have shown that DNMT3L affects the occurrence and development of gastric cancer through its anti-apoptotic effects [27], and plays a significant role in the growth of human embryonic tumors [28]. While it has been reported that hepatitis B virus X protein (HBx) may affect the occurrence of HCC through DNMT3L [29] and DNMT3L may regulate TNFRSF12A methylation, affecting the occurrence, development and prognosis of HCC [30], the specific mechanism by which DNMT3L contributes to the growth and metastasis of HCC remains unclear.

In this study, we combined analysis results from dozens of public datasets with basic experimental validation to reveal the role of DNMT3L in the growth and metastasis of HCC from multiple perspectives. Additionally, we discovered for the first time in HCC that DNMT3L can reduce the promoter methylation of cysteine dioxygenase 1 (CDO1) by competitively inhibiting DNMT3A, thereby exerting its anticancer function through an unconventional pathway that contradicts the prevailing view. Our study reveals a novel mechanism in the growth and metastasis of HCC and characterizes DNMT3L as a promising biomarker with diagnostic and therapeutic significance.

## Materials and methods

### In vitro cell proliferation

A density of 5000 cells/well was used to seed cells into 96-well plates, after which they were allowed to grow for 24 h. Next, 10 μl of Cell Counting Kit-8 (CCK-8) (Vazyme, Nanjng, China) was introduced to each well, followed by a 2-h incubation at 37 °C. The Eon™ Microplate Reader (BioTek, VT, USA) was used to determine the absorbance of each well at a wavelength of 450 nm.

### In vitro cell cycle assays

To evaluate the cell cycle, PI (Propidium, Iodide) staining assays were conducted. Cells were seeded in 96-well plates at a density of 5000 cells per well. Following 24 h of incubation, the cells were washed with PBS, fixed using 70% ice-cold ethanol and stained with PBS that contained 0.2 mM/ml PI (Sigma-Aldrich, Germany) and 0.2 mg/ml RNase (Yeasen, Shanghai, China). After an hour of incubation at 37 °C, the cells were scanned using the Acumen® eX3 (TTP LabTech, Melbourne, UK) with fluorescence signal excited at 488 nm. The cell cycle was then analyzed based on the scanning results.

### In vitro cell apoptosis assays

Hoechst-PI staining assays was used to assess cell apoptosis. Cells were seeded in 96-well plates at a density of 5000 cells per well and incubated for 24 h. After washing with PBS, cells were stained with 0.2 mM/ml PI (Sigma-Aldrich, Germany) and 10 μg/ml Hochest (bisBenzimide H33342, Sigma-Aldrich, Germany) in PBS for 20 min at 37 °C in the dark. The Acumen® eX3 (TTP LabTech, Melbourne, UK) was used to scan and analyze the cells.

### In vitro migration assays

For migration assays, cells were seeded at a density of 3 × 10^5^ or 8 × 10^5^ cells/well in 12 or 6-well plates. A sterile 10 μl pipette tip was used to create standardized wounds in confluent cell monolayers, which were then incubated for 24/48 h. The proportion of changed area was calculated using Image J (National Institutes of Health, USA).

### In vitro invasion assays

100 µl Matrigel matrix (Corning, USA) diluted in a 30:1 ratio with serum-free DMEM medium was added to the transwell chambers. 600 µl DMEM medium containing 10% FBS was added to the lower chamber and 5 × 10^5^ cells were plated in the upper chamber. After incubation at 37°C for 120 h (HCCLM3) or 96 h (Hep3B), the invaded cells were fixed with 4% paraformaldehyde and stained with Hochest (bisBenzimide H33342, Sigma-Aldrich, Germany) for 15 min in the dark.

### In vivo tumor growth and metastasis assays

The animal studies, which involved the use of male BALB/cA-Nu nude mice (BEIJING HFK BIOSCIENCE, Beijing, China) aged 6 weeks, were approved by the Animal Ethic Review Committees of the Second Affiliated Hospital of Zunyi Medical University. The mice were housed in standard pathogen-free conditions and were injected with matrigel containing 1 × 10^6^ cells to construct orthotopic implanted models for liver tumor growth and metastasis assays. Tail intravenous injection models (1 × 10^6^ cells) were established for lung tumor growth and metastasis assays. After four or six weeks, the mice were euthanized and their livers or lungs were removed for analysis and H&E staining. All animal experiments were conducted in accordance with the NIH Guide for the Care and Use of Laboratory Animals.

### Methylation-specific PCR (MSP) and Sanger Sequencing

The sample DNA was extracted using the DNA extraction kit (DP304, Tiangen, Beijing, China) following the manufacturer’s instructions. The final volume of the sample was 50 μl with a concentration range of 93.3–261.2 ng/μl. Bisulfite conversion of sample DNA was performed with the EpiTect fast DNA bisulfite kit (Qiagen, Hilden, Germany) as described by the manufacturer. Primer pairs targeting the methylated (M-CDO1-promoter) or un-methylated (U-CDO1-promoter) region (CpG island–dense region) of CDO1 promoter (1500 bps upstream of the TSS) were designed using MethPrimer software (https://www.urogene.org/methprimer2/). The PCR conditions were set according to the instructions of the 2 × EpiArt HS Taq Master Mix (Vazyme Biotech, Nanjing, China), with 200 ng genomic DNA and an annealing temperature of 56 ℃ for 40 cycles. The products were identified using a 2.5% agarose gel and ethidium bromide staining after PCR. Furthermore, to further assess the methylation status of the GpC sites in the CDO1 promoter mentioned above, the cytosine-to-thymine ratio in the sample DNA after bisulfite conversion was analyzed. This analysis was conducted using Sanger sequencing carried out by Shanghai outdo biotech company (Shanghai, China).

Primers used in this study:

**Table:** 

Primer names	Sequences
Left M-CDO1-promoter	5′-TTTTAAGCGTTTAAAAGGGGTTC-3′
Right M-CDO1-promoter	5′-CCAACATTAAAATACCGAAACGTA-3′
Left U-CDO1-promoter	5′-TTTAAGTGTTTAAAAGGGGTTTGG-3′
Right U-CDO1-promoter	5'-CCCAACATTAAAATACCAAAACATA-3′

### Construction of luciferase reporter plasmids

The pGL4.17 vector (Tsingke, Beijing, China) was digested using the enzymes Kpn I (Takara, Shiga, Japan) and Xho I (Takara, Shiga, Japan). The CDO1 promoter were amplified by PCR and inserted into the pGL4.17 vector using the ClonExpress II One Step Cloning Kit (Vazyme, NanJing, China), following the manufacturer's instructions. Subsequently, the constructed plasmids were transformed into TOP10 competent cells (Sangon Biotech, Shanghai, China) for plasmid production. The plasmid sequences were confirmed by DNA sequencing.

Primers used in this study:

**Table:** 

Primer names	Sequences
CDO1-promoter forward	5′-CTAACTGGCCGGTACCGCTGGCTTCTGGA-3′
CDO1-promoter reverse	5'-TCTTGATATCCTCGAGCTCGTGGGGAGC-3′

### Luciferase reporter assays

To assess the activity of the CDO1 promoter, HEK293T cells were co-transfected with pGL4.17-CDO1-promoter, OE-DNMT3A, OE-DNMT3L, or the control using the X-tremeGENE DNA transfection reagent (Roche, Indianapolis, IN, USA). After 48 h of transfection, luciferase activity was quantified using the Dualucif™ Firefly & Renilla Assay Kit (UElandy, Suzhou, China) following the manufacturer's instructions.

### Statistical analysis

The experimental data were presented as the mean ± standard error of the mean (SEM) and were obtained from three independent experiments. Statistical analysis was performed using GraphPad Prism 9 software. Student’s t-test or a nonparametric test was used to detect differences between two groups. Statistical significance was determined by P values less than 0.05. *P < 0.05, **P < 0.01, ***P < 0.001. Additional materials and methods are described in Additional file 1.

## Results

### DNMT3L is downregulated in HCC and is associated with poor prognosis

**Fig. 1:**
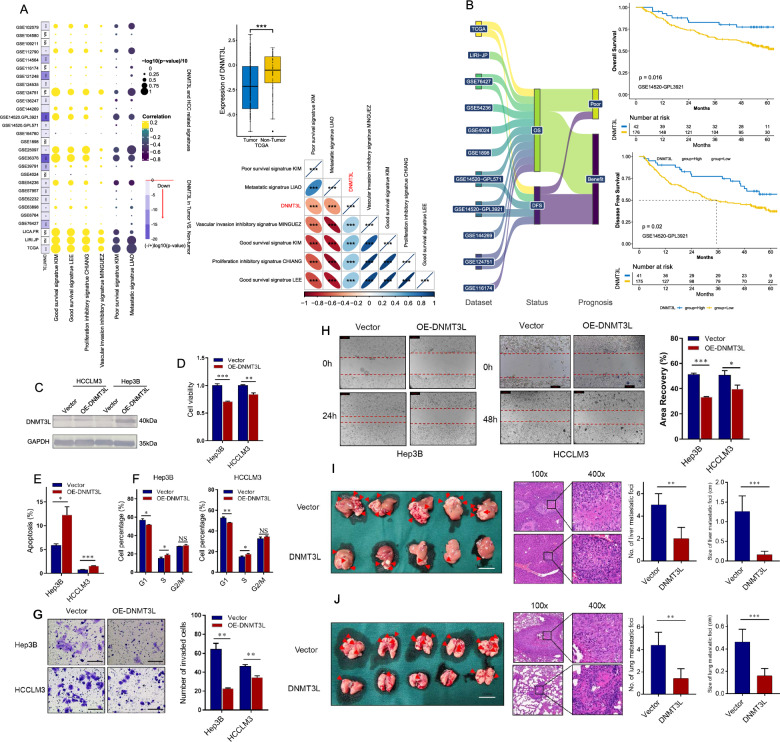
DNMT3L predicts well prognosis of HCC patients and inhibits tumor progression. **A** DNMT3L expression in HCC and non-tumor tissues and its correlation with HCC signatures in different datasets. Down, low expression in HCC tissues. **B** Correlation of DNMT3L expression with overall survival and disease-free survival in 11 datasets. The X-tile method was used to find the best cutoff value. **C** Plasmid transfection up-regulates DNMT3L expression in HCCLM3 and Hep3B cells. **D** CCK-8 assays for HCCLM3 and Hep3B cells transfected with OE-DNMT3L or the control. **E** Cell apoptosis was measured by Hoechst-PI staining assays in HCCLM3 and Hep3B cells transfected with OE-DNMT3L or the control. **F** Cell cycle analysis using PI staining in HCCLM3 and Hep3B cells transfected with OE-DNMT3L or the control. **G** Transwell invasion assays for HCCLM3 and Hep3B cells transfected with OE-DNMT3L or the control. Scale bars = 100 μm. **H** Wound-healing migration assays for HCCLM3 and Hep3B cells transfected with OE-DNMT3L or the control. Scale bars = 200 μm. **I** Intrahepatic metastatic nodules in orthotopic implantation models with indicated HCCLM3 cells. Scale bars = 20 mm. **J** Pulmonary metastatic nodules in lung metastasis models with indicated Hep3B and HCCLM3 cells. Scale bars = 10 mm. Data are presented as mean ± SEM. *P < 0.05, **P < 0.01, ***P < 0.001. NA: not available

To investigate the expression of DNMT3L in HCC and enhance the reliability of our findings, we conducted a comprehensive literature search and collected 31 publicly available datasets. Out of these datasets, 20 contained both HCC and non-tumor samples, along with corresponding DNMT3L expression values (Additional file 14: Table S1). The results demonstrated a consistent downregulation of DNMT3L expression in HCC across all 20 datasets, with a statistically significant reduction observed in 19 of them (p < 0.05) (Fig. 1A and Additional file 2: Fig. S1). Tumor development is an intricate process, and the emergence of tumor characteristics should be attributed to the aberration of multiple genes, rather than just one or a few factors [31]. Therefore, we collected six gene sets related to HCC proliferation, vascular invasion, metastasis, and prognosis, each containing dozens to hundreds of genes associated with the corresponding feature (Additional file 15: Table S2). We then used the GSVA method to quantify these gene sets as enrichment score (ES) and reflect the clinical features of the tumors [32, 33], which were referred to as HCC-related signatures. The results showed that among the 28 analyzable datasets, DNMT3L was predominantly positively associated with signatures indicating proliferation inhibition, vascular invasion inhibition, and good survival, while being negatively correlated with signatures indicating metastasis and poor survival (Fig. 1A and Additional file 3: Fig. S2). Additionally, we performed Kaplan–Meier analysis on 11 datasets containing both DNMT3L expression and prognosis information, and discovered that high DNMT3L expression was associated with longer overall survival (OS) and disease-free survival (DFS) in the majority of cases (Fig. 1B and Additional file 4: Fig. S3). Based on the above data analysis, it is suggested that DNMT3L may have an anti-tumor function in HCC.

### DNMT3L inhibits the tumor growth and metastasis

The aforementioned data analysis indicated that DNMT3L was downregulated in HCC and had a notable correlation with proliferation, vascular invasion, metastasis, and prognosis indicators, suggesting a potential anti-tumor function. To validate the analysis results, we conducted in vitro and in vivo experiments.

We examined the expression of DNMT3L in four human hepatoma cell lines and observed that it was relatively low in HCCLM3 and Hep3B (Additional file 13: Fig. S12A). Subsequently, we upregulated the expression of DNMT3L in HCCLM3 and Hep3B (Fig. 1C), and found that DNMT3L overexpression significantly inhibited cell proliferation, while leading to S-phase arrest and promoting apoptosis (Fig. 1D–F). Additionally, upregulation of DNMT3L significantly suppressed the invasive and migratory abilities of HCCLM3 and Hep3B cells (Fig. 1G, H). To further explore the tumorigenic role of DNMT3L in vivo, HCCLM3 cells infected with LV-DNMT3L or LV-Ctrl were used to establish liver orthotopic-implanted models and lung metastasis models in nude mice. In comparison to the control group, the LV-DNMT3L group demonstrated significantly smaller liver and lung tumors, as well as a lower incidence of liver and lung metastases (Fig. 1I, J). These findings further demonstrate that DNMT3L can inhibit tumor growth and metastasis in HCC.

### DNMT3L regulates CDO1 expression and promoter methylation

**Fig. 2:**
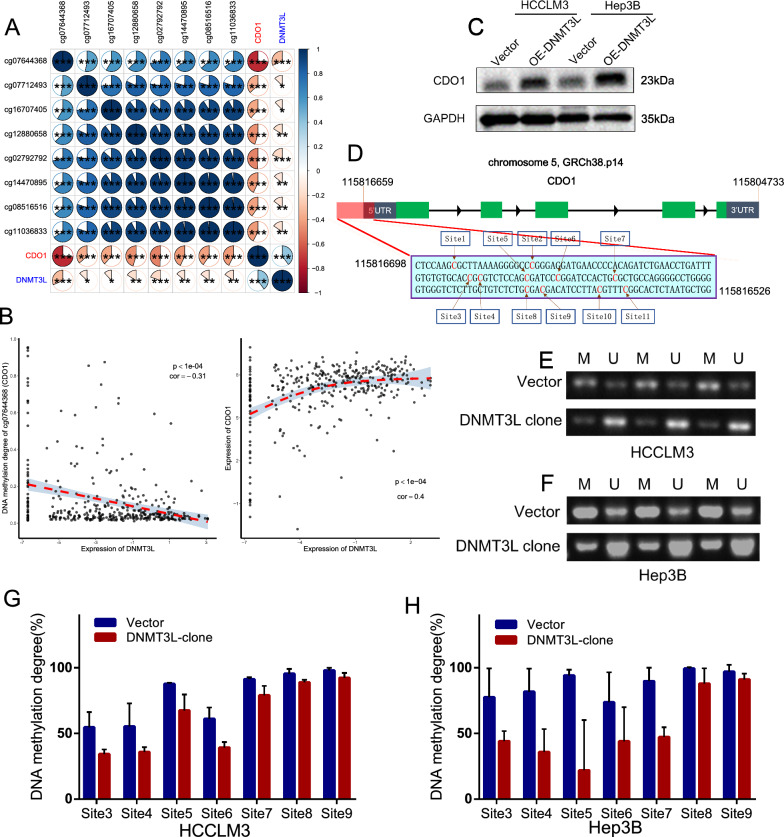
DNMT3L regulates CDO1 expression and promoter methylation. **A**, **B** Correlation of DNMT3L expression with CDO1 expression and promoter methylation in TCGA. **C** CpG island dense region in CDO1 promoter. **D** Western-blot analysis of CDO1 expression in HCCLM3 and Hep3B cells transfected with OE-DNMT3L or the control. **E**, **F** MSP test of CDO1 promoter in HCCLM3 and Hep3B cells transfected with OE-DNMT3L or the control. **G**, **H** Sanger sequencing after bisulfite conversion to analyze the methylation of CDO1 promoter in HCCLM3 and Hep3B cells transfected with OE-DNMT3L or the control

Since DNMT3L plays a crucial role in regulating DNA methylation, we analyzed its effects on DNA methylation by combining data from TCGA transcriptome and DNA methylation chips. To our surprise, we observed a significant decrease in methylation levels across different DNA regions in the high-DNMT3L group (Additional file 5: Fig. S4A). This discovery contradicts the widely held belief that DNMT3L promotes DNA methylation [25, 26]. After observing this unexpected phenomenon, we revisited studies related to DNMT3L and discovered that Neri et al. [34] also reported a similar situation. They found that in embryonic stem cells of mice with Dnmt3l knocked out, there was a significant increase in the methylation of bivalent gene promoters, suggesting that Dnmt3l can also inhibit DNA methylation. Consequently, we postulated that DNMT3L might exert its anti-cancer function by inhibiting DNA methylation. To explore this possibility, we analyzed the differential methylation sites (DMSs) of promoter and differential expressed genes (DEGs) between high and low-DNMT3L groups in TCGA, and identified a total of 225 DEGs and 206 DMSs on 143 genes (Additional file 5: Fig. S4B, S4C and Additional file 16: Table S3, Additional file 17: Table S4). After taking the intersection, we found three downstream candidate genes that may be regulated by DNMT3L-mediated methylation (Additional file 5: Fig. S4D). Given the positive correlation between DNMT3L and CDO1 expression, as well as the negative correlation between DNMT3L and methylation of multiple sites on the CDO1 promoter (Fig. 2A, B), we chose CDO1 as a downstream target gene of DNMT3L and carried out further validation.

CDO1 is a non-heme iron-containing enzyme mainly involved in the regulation of cysteine and the synthesis of sulfuric acid. Previous research has indicated that CDO1 is frequently silenced in tumors due to the high methylation of its promoter [35], and is associated with tumor chemo-sensitivity and prognosis [36, 37]. We conducted WB experiments and found that overexpression of DNMT3L led to an increase in CDO1 expression in both HCCLM3 and Hep3B cells (Fig. 2C). Additionally, we performed MSP experiments targeting CpG island-enriched regions of the CDO1 promoter (Fig. 2D). The results demonstrated that DNMT3L overexpression significantly inhibited methylation of the CDO1 promoter in HCCLM3 and Hep3B cells (Fig. 2E, F). Moreover, Sanger sequencing after bisulfite conversion suggested that overexpression of DNMT3L can lead to a decrease in methylation levels at multiple sites on the CDO1 promoter (Fig. 2G, H). These findings further support that DNMT3L can inhibit methylation of the CDO1 promoter and promote CDO1 expression.

### CDO1 is downregulated in HCC and is associated with poor prognosis

**Fig. 3:**
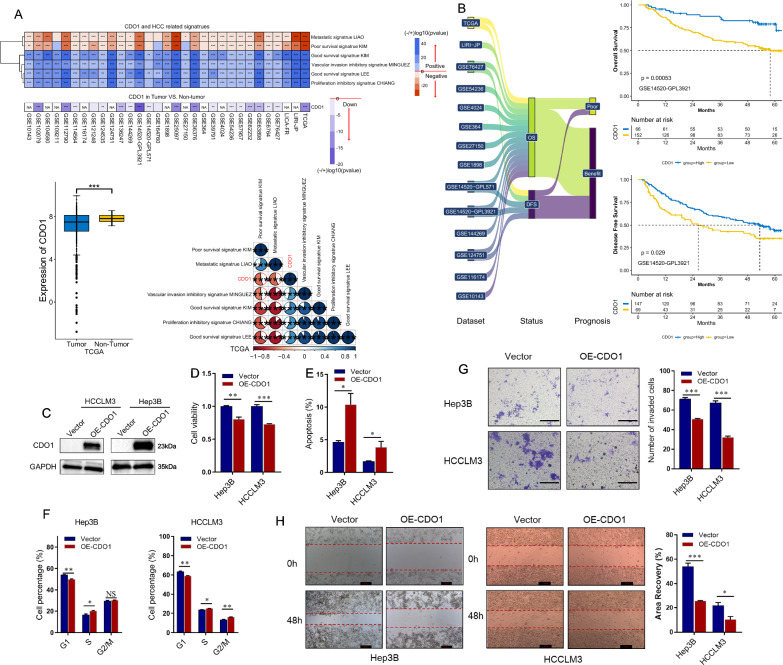
CDO1 predicts well prognosis of HCC patients and inhibits tumor progression. **A** CDO1 expression in HCC and non-tumor tissues and its correlation with HCC signatures in different datasets. Down, low expression in HCC tissues. **B** Correlation of CDO1 expression with overall survival and disease-free survival in 14 datasets. The X-tile method was used to find the best cutoff value. **C** Plasmid transfection up-regulates CDO1 expression in HCCLM3 and Hep3B cells. **D** CCK-8 assays for HCCLM3 and Hep3B cells transfected with OE-CDO1 or the control. **E** Cell apoptosis was measured by Hoechst-PI staining assays in HCCLM3 and Hep3B cells transfected with OE-CDO1 or the control. **F** Cell cycle analysis using PI staining in HCCLM3 and Hep3B cells transfected with OE-CDO1 or the control. **G** Transwell invasion assays for HCCLM3 and Hep3B cells transfected with OE-CDO1 or the control. Scale bars = 100 μm. **H** Wound-healing migration assays for HCCLM3 and Hep3B cells transfected with OE-CDO1 or the control. Scale bars = 200 μm. Data are presented as mean ± SEM. *P < 0.05, **P < 0.01, ***P < 0.001. NA: not available

Given that DNMT3L can inhibit methylation and promote expression of CDO1, we hypothesized that its anti-cancer role might be mediated through CDO1. If this hypothesis is true, CDO1, as a downstream regulatory gene, is also expected to exhibit anti-cancer functions. Consequently, we analyzed CDO1 in 31 publicly available datasets using the same methods. As expected, CDO1 was downregulated in tumors across all 20 analyzable datasets, with statistical significance observed in 18 of them (p < 0.05) (Fig. 3A and Additional file 6: Fig. S5). Across all 31 datasets, we observed a negative correlation between CDO1 expression and metastasis and poor survival signals, and a positive correlation with proliferation inhibition, vascular invasion inhibition, and good survival signals (Fig. 3A and Additional file 7: Fig. S6). Additionally, survival analysis on 14 analyzable datasets discovered that high CDO1 expression was associated with longer OS and DFS in the majority of cases (Fig. 3B and Additional file 8: Fig. S7).

### CDO1 suppresses the tumor growth and metastasis

To further investigate the potential anti-cancer effect of CDO1, we conducted in vivo functional experiments. Initially, we evaluated the expression of CDO1 in four human hepatoma cell lines and found that HCCLM3 and Hep3B had relatively low CDO1 expression levels similar to DNMT3L (Additional file 13: Fig. S12A). Subsequently, we upregulated the expression of CDO1 in HCCLM3 and Hep3B cells (Fig. 3C), and the results demonstrated that overexpression of CDO1 significantly inhibited cell proliferation and leaded to S-phase arrest, while promoting apoptosis (Fig. 3D–F). Furthermore, we observed that upregulation of CDO1 also significantly suppressed the invasive and migratory abilities of HCCLM3 and Hep3B cells (Fig. 3G, H). These experimental findings provide additional evidence for the potential of CDO1 as an inhibitory factor for tumor growth and metastasis in HCC.

To gain further insights into the potential mechanisms of CDO1 in HCC, we conducted GSVA analysis on 31 datasets to explore the association between CDO1 and Hallmark gene sets (Additional file 18: Table S5). The results revealed a significant positive correlation between CDO1 and metabolic pathways, including bile acid, fatty acid, and xenobiotic metabolism in HCC. Moreover, CDO1 exhibited the role to suppress the G2M checkpoint and mitotic spindle signaling (Additional file 9: Fig. S8A, B). Additionally, we identified the top 200 genes that exhibited the strongest correlation with CDO1 across the 31 HCC datasets (Additional file 19: Table S6, Additional file 9: Fig. S8C). KEGG analysis demonstrated that these genes were predominantly enriched in pathways such as glycolysis, tyrosine, pyruvate, and carbon metabolism, and fatty acid degradation (Additional file 20: Table S7, Additional file 9: Fig. S8D). GO analysis indicated that these genes were primarily involved in biological processes such as lipid and carboxylic catabolism, triglyceride, fatty acid, and small molecular metabolism (Additional file 21: Table S8, Additional file 9: Fig. S8E). Collectively, these analytical findings further support the regulatory role of CDO1 in the cell cycle of HCC and suggest its potential significance in tumor metabolism.

### DNMT3L suppresses the tumor growth and metastasis through CDO1

**Fig. 4:**
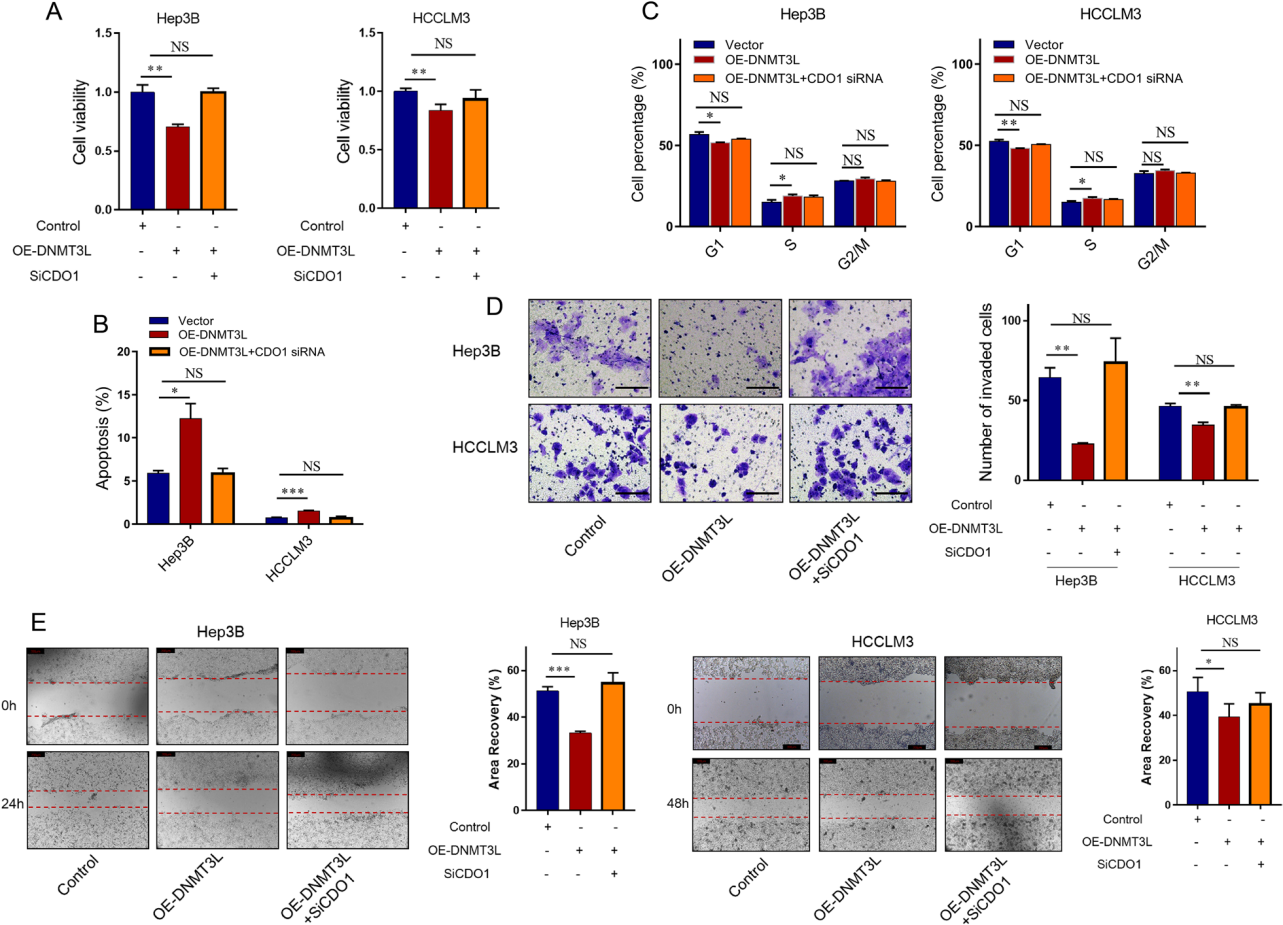
DNMT3L mediates the cell proliferation and invasion through CDO1. **A** CCK-8 assays for indicated cells. **B** Cell apoptosis was measured by Hoechst-PI staining assays for indicated cells. **C** Cell cycle analysis using PI staining in indicated cells. **D** Transwell invasion assays for indicated cells. Scale bars = 100 μm. **E** Wound-healing migration assays for indicated cells. Scale bars = 200 μm. Data are presented as mean ± SEM. *P < 0.05, **P < 0.01, ***P < 0.001. NS: no significant

Based on our previous data analysis and experimental validation, we have confirmed the anti-tumor function of DNMT3L and CDO1, and have demonstrated that DNMT3L can regulate both the DNA methylation and expression of CDO1. To explore whether DNMT3L regulates the malignant cell phenotype of HCC through CDO1, we assessed whether inhibiting CDO1 could reverse the effects of DNMT3L overexpression on the biological behaviors of hepatoma cells. Specifically, we treated DNMT3L-overexpressing cells with siRNAs (siRNA1, Additional file 13: Fig. S12B) targeting CDO1. CCK8 and PI staining assays showed that DNMT3L overexpression significantly inhibited cell proliferation and cell cycle, while further knockdown of CDO1 by siRNA rescued the inhibition caused by DNMT3L (Fig. 4A, C). Overexpression of DNMT3L promoted cell apoptosis, and further downregulation of CDO1 restrained this promotion (Fig. 4B). Trans-well invasion and wound-healing migration assays demonstrated that knocking down CDO1 by siRNA in HCCLM3 and Hep3B cells reversed the invasive and migratory inhibition caused by the upregulation of DNMT3L (Fig. 4D, E). These results indicate that CDO1 mediates the tumor growth and metastasis inhibition caused by DNMT3L.

### DNMT3L competitively inhibits the regulation of CDO1 by DNMT3A

**Fig. 5:**
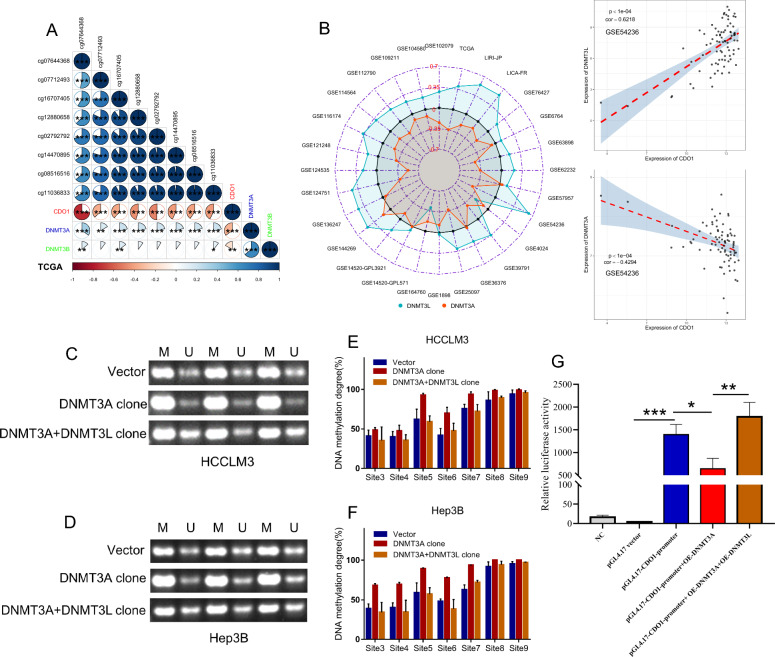
DNMT3L affects the regulation of DNMT3A on CDO1. **A** Correlation of DNMT3A/DNMT3B expression with CDO1 expression and promoter methylation in TCGA. **B** Correlation of DNMT3L and DNMT3A with CDO1 in 28 datasets. **C**, **D** MSP test of CDO1 promoter in HCCLM3 and Hep3B cells transfected with OE-DNMT3A, OE-DNMT3A and OE-DNMT3L, or the control. **E**, **F** Sanger sequencing after bisulfite conversion analyze the methylation of CDO1 promoter in HCCLM3 and Hep3B cells transfected with OE-DNMT3A, OE-DNMT3A and OE-DNMT3L, or the control. **G** Relative luciferase activity in HEK293T cells co-transfected with luciferase reporter pGL4.17-CDO1-promoter, OE-DNMT3A, OE-DNMT3L, or the control. *P < 0.05, **P < 0.01, ***P < 0.001

As DNMT3L does not possess DNA methylation catalytic activity and there are no reports indicating that it can reverse DNA methylation like the TET family proteins, we started to ponder the potential mechanism by which DNMT3L inhibits the methylation of the CDO1 promoter. Neri et al. [34] have reported that in mouse embryonic stem cells, Dnmt3l can compete with Dnmt3a and Dnmt3b to inhibit the promoter methylation of Rhox5, which gave us inspiration. In the analysis of TCGA, we observed a positive correlation between both DNMT3A and DNMT3B with the methylation of the CDO1 promoter, while a negative correlation was observed between the expression of CDO1 and both DNMT3A and DNMT3B (Fig. 5A). Given that the effect of DNMT3A was more significant than DNMT3B and there was a significant increase in methylation levels across different DNA regions in the high-DNMT3A group (Additional file 10: Fig. S9), we chose to focus our further analysis on DNMT3A. Subsequently, we examined multiple analyzable datasets and observed that DNMT3L is primarily positively correlated with CDO1 expression, while DNMT3A is primarily negatively correlated with CDO1 expression (Fig. 5B and Additional file 11: Fig. S10, Additional file 12: Fig. S11), which further supports our hypothesis.

To further confirm our hypothesis, we upregulated the expression of DNMT3A in both HCCLM3 and Hep3B (Additional file 13: Fig. S12C). MSP analysis revealed that overexpression of DNMT3A significantly increased methylation of the CDO1 promoter, while upregulation of DNMT3L reversed the effect of DNMT3A on the CDO1 promoter (Fig. 5C, D). Sanger sequencing after bisulfite conversion also demonstrated consistent findings (Fig. 5E, F). These results indicate that DNMT3L can inhibit the pro-methylation effect of DNMT3A on the CDO1 promoter. In addition, we synthesized a pGL4.17 luciferase reporter plasmid containing the wild-type CDO1 promoter. The fluorescence activity of pGL4.17-CDO1-promoter showed a marked decrease upon DNMT3A overexpression, while upregulation of DNMT3L reversed this effect (Fig. 5G). These observations suggest that DNMT3A regulates the expression of CDO1 through its promoter, and DNMT3L can inhibit the regulatory effect of DNMT3A on the CDO1 promoter. Taken together, these experimental results demonstrate that DNMT3L can promote the expression of CDO1 by competitively inhibiting DNMT3A's methylation regulation on the CDO1 promoter.

## Discussion

With the arrival of the big data era, the use of statistical, machine learning, data mining, and other techniques to process and analyze large amounts of data has the potential to uncover patterns and trends hidden within the data, thus playing a key role in guiding basic scientific research [38, 39]. In comparison to traditional research, which often relies on limited data, big data analysis enables a thorough exploration of vast datasets, potentially leading to a more comprehensive understanding of the nature and characteristics of various phenomena. In the field of oncology, the emergence of high-throughput sequencing, gene chips, and other technologies has resulted in explosive growth in tumor omics data. Over the past two decades, tumor research has gradually transitioned into the era of big data analysis, thereby driving the in-depth development of this field. However, tumor omics data has its own unique characteristics. For instance, when examining tumor genomics data, whether through RNA sequencing or gene chips, there are still issues with accuracy and repeatability due to factors such as sample quality, RNA or DNA extraction and purification processes, detection platform selection, different batch processing, and other variables [40–42]. Additionally, due to limitations such as the number of tumor patients, medical ethics, laws and regulations, and technical difficulties, the sample size of a single-center, single-disease is typically small, which leads to potential sample selection bias and makes it difficult to represent the entire patient population. Therefore, a multi-center large-sample data analysis model is particularly crucial in tumor research, as it offers more reliable results [2]. As a widespread epigenetic modification in the eukaryotic genome, DNA methylation has obvious pathological significance in human diseases, especially in cancer [43, 44]. For DNA methylation regulatory factors, the specific functions and molecular mechanisms of DNMT1, DNMT3A, DNMT3B, and TET family proteins have been extensively studied in various malignant tumors [22–24, 45]. However, research on DNMT3L in cancer is very limited, and its role and mechanism are still largely unclear. Therefore, our study focuses on DNMT3L, gathering over 30 public HCC datasets with more than 5000 samples. Our objective is to use big data analysis to guide experiments to uncover the biological functions and potential mechanisms of DNMT3L in HCC, and to demonstrate the feasibility of conducting basic research with the help of big data.

In the specific research process, we employed big data analysis to investigate the association of DNMT3L with prognostic, proliferative, metastatic, and invasive signaling across over 30 HCC datasets, deducing its biological role in HCC. Big data analysis revealed that DNMT3L expression was consistently lower in HCC tissues and showed a significant correlation with favorable prognosis. Furthermore, DNMT3L was associated with inhibition signals of tumor metastasis, invasion, and proliferation. These trends were broadly consistent across most datasets (Fig. 1A, B). Given that big data analysis typically only offers correlational information rather than direct causal relationships [46], we consequently employed experiments to verify these findings. Further functional experiments indicated that DNMT3L could inhibit cell cycle progression, proliferation, apoptosis resistance, invasion, and migration in vitro, as well as suppress tumor growth and metastasis in vivo (Fig. 1C–G), which is consistent with the findings of big data analysis. Besides, we delved into the regulatory mechanisms of DNMT3L on proliferation and metastasis through an integrative strategy that combines big data analysis with basic experiments. Given that DNMT3L is a crucial DNA methylation regulator, we utilized methylation and transcriptome data to identify downstream regulatory factors of DNMT3L and discovered that CDO1 is likely regulated by DNMT3L-mediated methylation (Fig. 2A, B). Through Sanger sequencing after bisulfite conversion, MSP, and western blotting, we confirmed the accuracy of the data analysis results, showing that DNMT3L could inhibit methylation of the CDO1 promoter and increase its expression (Fig. 2C–H). Moreover, through the integration of big data analysis and experimental validation, we have clarified the anti-tumor activity of CDO1 in HCC (Fig. 3), and the rescue experiments have shown that DNMT3L can effectively suppress tumor growth and metastasis by regulating CDO1 (Fig. 4). Previous study has reported that DNMT3L is a catalytically inactive regulatory factor [47], which, unlike the TET family proteins, is incapable of directly reversing DNA methylation. Neri et al. [34] have discovered that Dnmt3l can inhibit promoter methylation by competing with Dnmt3a in mice. Therefore, we further conducted comparative analysis using big data and found that DNMT3A is significantly positively correlated with the methylation of the CDO1 promoter, and negatively correlated with CDO1 expression in most datasets. In stark contrast, the effect of DNMT3L on CDO1 is almost completely opposite, which suggests that these two factors may compete with each other to regulate CDO1 expression (Fig. 5A, B). Through MSP, Sanger sequencing after bisulfite conversion, and dual-luciferase assays, we further confirmed that DNMT3L can reverse DNMT3A-mediated promoter methylation and transcriptional regulation of CDO1 (Fig. 5C–G). Overall, our research has progressively uncovered the function and molecular mechanisms of DNMT3L in HCC through big data analysis and experimental validation. The study also suggests that big data analysis can effectively direct basic research, and in turn, the implementation of basic research can further validate the reliability and accuracy of big data analysis.

We also encountered an unexpected finding in this study. In previous research, DNMT3L has been commonly considered a cofactor which could promote DNA methylation by recruiting or activating DNMT3A [25, 48, 49]. In contrast, our study revealed that DNMT3L has an inhibitory effect on DNA methylation of the CDO1 promoter, which seems to contradict the mainstream view. However, Neri et al.'s study [34] confirmed that Dnmt3l can also inhibit methylation of gene promoters in mice, which is similar to our research. This phenomenon suggests that the DNA methylation inhibitory effect of DNMT3L may be a characteristic shared among species, rather than an isolated occurrence. Moreover, these studies indicate that the role of DNMT3L is more complex than previously understood. Beyond its established role in enhancing DNA methylation, DNMT3L could exhibit an "unconventional" mechanism that suppresses methylation. In this study, our findings demonstrated that DNMT3L could inhibit CDO1 promoter methylation. Additionally, our data analysis identified a positive correlation between DNMT3L levels and ITGB8 promoter methylation, inversely correlating with ITGB8 expression (Additional file 5: Fig. S4D, Additional file 16: Table S3, Additional file 17: Table S4). ITGB8 has been reported to have a pro-oncogenic role in tumors [50, 51]. Therefore, DNMT3L may inhibit the oncogenic activity of ITGB8 by promoting its promoter methylation. This implies a potential dual function of DNMT3L in HCC. However, further experimental validation is required to confirm this hypothesis. While these insights deepen our understanding of DNA methylation regulatory mechanisms, they also bring new questions and challenges. Key among these are the specific conditions under which DNMT3L could promote or inhibit DNA methylation. For example, does DNMT3L only inhibit the promoter methylation of specific genes? And if so, do these genes share common characteristics? Addressing these queries will require more comprehensive investigation.

Certainly, this study has some limitations. Firstly, the accuracy and reliability of big data analysis heavily rely on the availability of reliable data sources. In this study, publicly available datasets were utilized, which limits our control over the quality of the data. Additionally, second-generation sequencing and gene chip technologies possess inherent limitations in terms of accuracy and repeatability. Even when the same sample is analyzed using the same technology, variations in the results can occur. Moreover, the complexity of big data has spawned numerous analysis methods, and different methods may yield diverse outcomes. Therefore, it is necessary to establish standardized and scientifically rigorous procedures during sample detection and collection to ensure data quality and accuracy, and it is a need to develop more precise high-throughput detection technologies and efficient analysis methods to enhance the reliability of the results. Secondly, this study exclusively explored the potential mechanisms of CDO1 in HCC through data analysis, proposing that CDO1 may exert its role in HCC by regulating tumor metabolism. However, further experimental validation has not been conducted. This aspect will be a focal point of our future research (Additional file 1).

## Conclusion

**Fig. 6:**
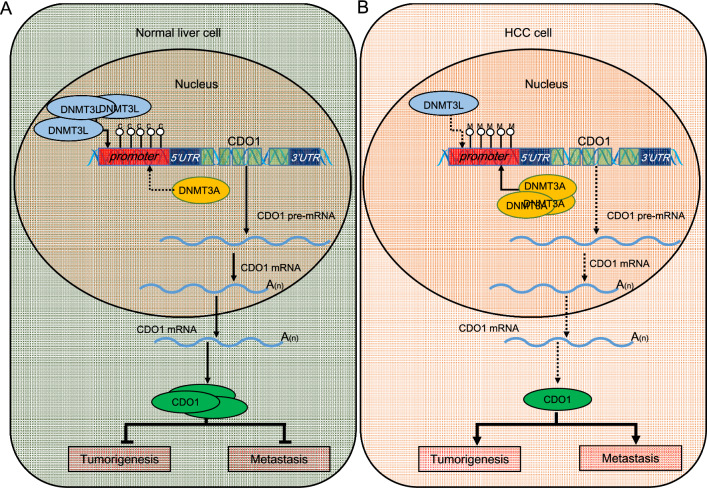
Mechanistic diagram of the epi-transcriptomic regulation underlying the DNMT3L-DNMT3A-CDO1 pathway. **A** Mechanistic diagram in normal liver cells. **B** Mechanistic diagram in HCC cells

In this study, we have uncovered the epi-transcriptomic regulatory mechanism of the DNMT3L-DNMT3A-CDO1 pathway. Specifically, under normal conditions, DNMT3L competitively inhibits DNMT3A's pro-methylation effect on the CDO1 promoter, leading to a relatively high expression of CDO1 (Fig. 6A). Conversely, when DNMT3L expression is abnormal, DNMT3A can facilitate the methylation of CDO1 promoter, thus downregulating CDO1 expression and promoting tumor growth and metastasis (Fig. 6B). Furthermore, almost every experiment in this study was supported by big data analysis, which enabled us to precisely define the research direction. The congruence between the analytical guidance and experimental verification highlighted the essential role and applicability of big data analysis in elucidating complex biological processes.

### Supplementary Information

**Additional file 1:** Supplementary materials and methods.

**Additional file 2: Figure S1.** DNMT3L expression in HCC and non-tumor tissues in 20 datasets. *P < 0.05, **P < 0.01, ***P < 0.001. NS, no significant.

**Additional file 3: Figure S2.** Correlations between DNMT3L expression and HCC signatures in 28 datasets. *P < 0.05, **P < 0.01, ***P < 0.001.

**Additional file 4:** **Figure S3.** The relationship between DNMT3L expression and overall survival or disease-free survival in 11 datasets.

**Additional file 5:** **Figure S4.** Screening of downstream target of DNMT3L. **A,** The relationship between DNMT3L expression and DNA methylation in TCGA. **B,** Volcano and heat-map plots show DEGs between DNMT3L-low and high groups in TCGA. **C,** Volcano plot show DMSs between DNMT3L-low and high groups in TCGA. **D,** Schematic of the selection for the downstream target of DNMT3L. TSS, transcription start site. TSS200: 0–200 bps downstream of TSS. TSS1500: 200–1500 bps downstream of TSS. TSS-200: 0–200 bps upstream of TSS. TSS-1500: 200–1500 bps upstream of TSS. TSSMT1500, more than 1500 bps from TSS. ALL, the whole DNA fragment. Island, CpG islands. N-Shelf, 2–4 kb upstream of CpG islands. S-Shelf, 2–4 kb downstream of CpG islands. N-Shore, 0–2 kb upstream of CpG islands. S-Shore, 0–2 kb downstream of CpG islands. Other: sites not on CpG islands, shelf and shore regions. DEGs, differential expressed genes. DMSs, differential methylation sites. *P < 0.05, **P < 0.01.

**Additional file 6:** **Figure S5.** CDO1 expression in HCC and non-tumor tissues in 20 datasets. *P < 0.05, **P < 0.01, ***P < 0.001. NS, no significant.

**Additional file 7:** **Figure S6.** The relationship between CDO1 expression and HCC signatures in 31 datasets. *P < 0.05, **P < 0.01, ***P < 0.001.

**Additional file 8:** **Figure S7.** The relationship between CDO1 expression and overall survival or disease-free survival in 14 datasets.

**Additional file 9:** **Figure S8.** GSVA, KEGG and GO analysis of CDO1. **A,** Relationship between CDO1 and hallmark gene sets. **B,** Correlation of CDO1 with bile acid metabolism, fatty acid metabolism, xenobiotic metabolism, and G2M checkpoint in TCGA. **C,** The top 200 genes with the highest correlation to CDO1. **D,** The top 10 KEGG enrichment-pathways associated with CDO1. **E**, The top 10 GO enrichment-pathways associated with CDO1. Cor, correlation coefficient. Negative, negative correlation. Positive, positive correlation.

**Additional file 10:** **Figure S9.** The relationship between DNMT3A expression and DNA methylation in TCGA. TSS, transcription start site. TSS200: 0–200 bps downstream of TSS. TSS1500: 200–1500 bps downstream of TSS. TSS-200: 0–200 bps upstream of TSS. TSS-1500: 200–1500 bps upstream of TSS. TSSMT1500, more than 1500 bps from TSS. ALL, the whole DNA fragment. Island, CpG islands. N-Shelf, 2–4 kb upstream of CpG islands. S-Shelf, 2–4 kb downstream of CpG islands. N-Shore, 0–2 kb upstream of CpG islands. S-Shore, 0–2 kb downstream of CpG islands. Other: sites not on CpG islands, shelf and shore regions. *P < 0.05, **P < 0.01.

**Additional file 11:** **Figure S10.** Correlations between DNMT3L expression and CDO1 expression in 28 datasets.

**Additional file 12:** **Figure S11.** The relationship between DNMT3A expression and CDO1 expression in 30 datasets.

**Additional file 13:** **Figure S12.** **A,** Western-blot analysis of DNMT3L and CDO1 expression in SNU449, SK-hep1, HCCLM3 and Hep3B cells. **B,** Western-blot analysis of CDO1 expression in HCCLM3 and Hep3B cells with transfected with CDO1 siRNA or the control. **C,** Plasmid transfection up-regulates DNMT3A expression in HCCLM3 and Hep3B cells.

**Additional file 14:** **Table S1.** Details of public datasets included in this study.

**Additional file 15:** **Table S2.** Details of HCC signatures included in this study.

**Additional file 16:** **Table S3.** DEGs between DNMT3L-low and high groups in TCGA. DEGs, differentially expressed genes. Up, up-regulated in HCC. Down, down-regulated in HCC.

**Additional file 17:** **Table S4.** DMSs between DNMT3L-low and high groups in TCGA. DMSs, differentially methylated sites. Up, up-regulated in HCC. Down, down-regulated in HCC.

**Additional file 18:** **Table S5.** Correlation between CDO1 and 50 hallmark gene sets in 31 datasets. Cor, correlation coefficient.

**Additional file 19:** **Table S6.** Correlation between CDO1 and other genes in 31 datasets. Cor, correlation coefficient.

**Additional file 20:** **Table S7.** KEGG analysis of the top 200 genes most associated with CDO1.

**Additional file 21:** **Table S8.** GO analysis of the top 200 genes most associated with CDO1.

## Data Availability

The datasets used in this article are mentioned in Additional file 14: Table S1 and are available in the GEO, ICGC, and TCGA public databases. The datasets used and analyzed during the current study are also available from the corresponding author on reasonable request.

## Abbreviations

HCC: Hepatocellular carcinoma
TACE: Transarterial chemoembolization
DNMTs: DNA methyltransferases
5mC: 5-Methylcytosine
DNMT3L: DNA methyltransferase 3-like
CDO1: Cysteine dioxygenase 1
CCK-8: Cell Counting Kit-8
MSP: Methylation-specific PCR
DMSs: Differential methylation sites
DEGs: Differential expressed genes
ES: Enrichment score
OS: Overall survival
DFS: Disease-free survival
TCGA: Genome Cancer Atlas
ICGC: International Cancer Genome Consortium
GEO: Gene Expression Omnibus
GSVA: Gene set variation analysis
ChAMP: Chip analysis methylation pipeline bio-conductor
GO: Gene Ontology
KEGG: Kyoto Encyclopedia of Genes and Genomes
