# Supplementary material for: DNMT3L inhibits hepatocellular carcinoma progression through DNA methylation of CDO1: insights from big data to basic research
Source: J Transl Med. 2024 Feb 2;22:128. doi: 10.1186/s12967-024-04939-9 (PMC10837993; doi:10.1186/s12967-024-04939-9)
Supplement: Supplementary file 1 — Additional file 1: Supplementary materials and methods. [file 12967_2024_4939_MOESM1_ESM.docx]

**Supplemental Materials and Methods**

**Acquisition of HCC datasets in TCGA, GEO, and ICGC**

In order to ensure the reliability of the analysis results, we searched the HCC datasets in the whole network. Finally, thirty-one HCC datasets with more than 20 HCC samples (Table S1) were downloaded from the Genome Cancer Atlas( TCGA，<https://portal.gdc.cancer.gov/>), International Cancer Genome Consortium (ICGC, <https://dcc.icgc.org/releases/current/Projects>), and Gene Expression Omnibus (GEO, <https://www.ncbi.nlm.nih.gov/geo>), respectively. The clinical information acquisition methods of above datasets are consistent with our previous research^1^. Expression profiling by high throughput sequencing were normalized with the TPM method. In datasets of expression profiling by array, probes and gene names matching and duplicate gene names processing methods were the same as previous research^2^. For each dataset, genes expressed in over 70% of samples were retained, and the missing values were replaced with the corresponding gene’s minimum value.

**Assessment of HCC-related signatures and hallmark gene sets**

Genes in six HCC-related signatures were obtained through literature review^3-7^, and table S2 contains more details about them. Hallmark gene sets were downloaded from the Molecular Signatures Database (MSigDB, <https://www.gsea-msigdb.org>). Both the HCC-related signatures and hallmark gene sets were assessed with the Gene Set Variation Analysis (GSVA) method^8^.

**DNA methylation degree assessment in 450k DNA methylation arrays**

DNA methylation level can be calculated by obtaining the average of different regions or total DNA fragments. [For example, the DNA methylation level of the TSS (transcription start site) upstream 200bp region can be calculated by obtaining all probes corresponding to this region through the manufacturer’s annotation file, summing up the beta values measured by each probe, and dividing by the total number of probes to obtain the methylation level of this region](https://academic.oup.com/nar/article/49/3/1313/6090302).

**Bioinformatics and clinical analysis**

Data processing, analysis and visualization were done with R (<https://www.r-project.org/>, v 4.1.0). Non-parametric Mann–Whitney U-test was used to detect differences of DNMT3L between HCCs and non-tumor tissues, and p < 0.05 (two-sided) was considered statistically significant. Correlation analysis was performed to evaluate the association of DNMT3L with CDO1 and six HCC-related signatures. Univariate Cox regression and log-rank test were used to analysis the impact of DNMT3L on patients’ outcome, and X-tile (version 3.6.1, Yale University School of Medicine) was used for selecting the best cut-off value. Differential expressed genes (DEGs) between DNMT3L-low and high groups were identified with limma method^9^, and p-value < 0.001 and |logFC (log fold change)|> 1.5 was set as the cut-off criteria. Chip Analysis Methylation Pipeline Bio-conductor (ChAMP) method was used to identify differential methylation sites (DMSs)^10^, while p-value < 0.001 and |logFC (log fold change)|> 0.9 was considered statistically significant. The relationship between CDO1 and tumor tissues, HCC-related signatures, hallmark gene sets, prognosis, methylation level and DNMT3A was evaluated with the same methods above. The R package clusterProfiler v4.6.2 was employed to carry out Gene Ontology (GO) and Kyoto Encyclopedia of Genes and Genomes (KEGG) analysis^11^ , aiming to explore the potential biological functions of CDO1. The enrichplot package was utilized for visualizing the primary GO terms and KEGG pathways.

**Cell lines**

SNU449, SK-hep1, HCCLM3, Hep3B and HEK293T cell lines were provided by Shanghai outdo biotech company (Shanghai, China). All cell lines were characterized by short tandem repeat (STR) analysis by Genetic Testing Biotechnology Corporation (Suzhou, China).

**Western blot analysis**

The cell lysates were prepared using RIPA Lysis Buffer (Beyotime Biotechnology, Shanghai, China), which was supplemented with a protease inhibitor cocktail (PMSF, Beyotime Biotechnology, Shanghai, China). The protein concentration was determined using the BCA Protein Assay Kit (Beyotime Biotechnology, Shanghai, China). The proteins were separated using sodium dodecyl sulfate-polyacrylamide gel electrophoresis (SDS-PAGE, Beyotime Biotechnology, Shanghai, China) and transferred onto PVDF membranes (Merck-Millipore, Massachusetts, USA). Following primary antibody incubation, the membranes were exposed to horseradish peroxidase or alkaline phosphatase-conjugated IgG secondary antibodies (Solarbio, Beijing, China; Beyotime Biotechnology, Shanghai, China). Immunoreactivity was detected using either the enhanced chemiluminescent (ECL) chromogenic substrate (Merck-Millipore, Massachusetts, USA) or BCIP/NBT alkaline phosphatase chromogenic kit (Beyotime Biotechnology, Shanghai, China). The ChemiDoc MP Imager System (Bio-Rad, California, USA) was utilized to scan the intensity of signals, which were then analyzed using Image Lab 5.2 software (Bio-Rad, California, USA).

Primary antibodies used in this study:

| Antigens | Manufacturer | Catalog Number | Application |
| --- | --- | --- | --- |
| DNMT3L  CDO1  GAPDH  DNMT3A | Abclonal  Proteintech  Proteintech  huabio | A13591  12589-1-AP  60004-1-Ig  ET1609-31 | 1:500 for WB  1:1000 for WB  1:20000 for WB  1: 1000 for WB |
| WB, western blot. | | | |

**Construction of plasmid, lentiviral packaging and RNA interference**

The human DNMT3L (NM_013369.4), DNMT3A (NM_153759.3), and CDO1 (NM_001323565.2) sequence was synthesized and inserted into the MCS (multiple cloning site) of pEX-2 vector by Shanghai Outdo Biotech Company (Shanghai, China) to produce OE-DNMT3L, OE-DNMT3A, or OE-CDO1, respectively. The recombinant product was then transformed into DH5α competent cells for clone using kanamycin selection and confirmed through PCR amplification and DNA sequencing. Lentiviral packaging of the plasmid overexpressing DNMT3L was performed by Beijing Augct biotechnology Co., Ltd. (Beijing, China), and viruses were produced in HEK293T cells. Shanghai Outdo Biotech Company (Shanghai, China) also synthesized small interfering RNA (siRNA) oligonucleotides targeting CDO1, as well as control siRNA.

Primers used in this study:

| Primer names | Sequences |
| --- | --- |
| CDO1 forward | 5'- CTACCGGACTCAGATCTCGAGATGGAACAGACCGAAGTG -3' |
| CDO1 reverse | 5'- GTACCGTCGACTGCAGAATTCTTAGTTGTTCTCCAGCGAG -3' |
| DNMT3L forward | 5'- CGGACTCAGATCTCGAGATGGCGGCCATCCC -3' |
| DNMT3L reverse | 5'- TACCGTCGACTGCAGAATTCTTATAAAGAGGAAGTGAGTT -3' |
| DNMT3A forward | 5'- AGCGCTACCGGACTCAGATCTATGGGGATCCTGGAGCGG -3' |
| DNMT3A reverse | 5'- GTACCGTCGACTGCAGAATTCTTACACACACGCAAAATACTCCTTC -3' |

Sequences of siRNA against specific target in this study:

|  | Sequences |
| --- | --- |
| CDO1 siRNA1 sense | 5'-CCGAAAUCUUGUGGAUCAATT-3' |
| CDO1 siRNA1 anti-sense | 5'-UUGAUCCACAAGAUUUCGGGT-3' |
| CDO1 siRNA2 sense | 5'-CCAGUGUGCCUACAUCAAUTT-3' |
| CDO1 siRNA2 anti-sense | 5'- AUUGAUGUAGGCACACUGGTT-3' |
| CDO1 siRNA3 sense | 5'-GGAAAUCUAAAGGAGACAUTT-3' |
| CDO1 siRNA3 anti-sense | 5'-AUGUCUCCUUUAGAUUUCCCT-3' |
| Control siRNA sense | 5'- UUCUCCGAACGAGUCACGUTT -3' |
| Control siRNA anti-sense | 5'- ACGUGACUCGUUCGGAGAATT-3' |

**Cell transfection**

1×10^6^ cells per well were transfected with siRNAs using X-tremeGENE siRNA transfection reagent (Roche, Indianapolis, IN, USA) according to the manufacturer’s protocol. X-tremeGENE HP DNA reagent (Roche, Indianapolis, IN, USA) was used for cell transfection with plasmids. The cells were harvested 24 h after transfection.

1. Liu X, Xiao C, Yue K, Chen M, Zhou H, Yan X. Identification of multi-omics biomarkers and construction of the novel prognostic model for hepatocellular carcinoma. *Scientific reports*. Jul 15 2022;12(1):12084. doi:10.1038/s41598-022-16341-w

2. Yan X, Wan H, Hao X, et al. Importance of gene expression signatures in pancreatic cancer prognosis and the establishment of a prediction model. *Cancer management and research*. 2019;11:273-283. doi:10.2147/cmar.S185205

3. Kim SM, Leem SH, Chu IS, et al. Sixty-five gene-based risk score classifier predicts overall survival in hepatocellular carcinoma. *Hepatology (Baltimore, Md)*. May 2012;55(5):1443-52. doi:10.1002/hep.24813

4. Chiang DY, Villanueva A, Hoshida Y, et al. Focal gains of VEGFA and molecular classification of hepatocellular carcinoma. *Cancer research*. Aug 15 2008;68(16):6779-88. doi:10.1158/0008-5472.Can-08-0742

5. Lee JS, Chu IS, Heo J, et al. Classification and prediction of survival in hepatocellular carcinoma by gene expression profiling. *Hepatology (Baltimore, Md)*. Sep 2004;40(3):667-76. doi:10.1002/hep.20375

6. Liao YL, Sun YM, Chau GY, et al. Identification of SOX4 target genes using phylogenetic footprinting-based prediction from expression microarrays suggests that overexpression of SOX4 potentiates metastasis in hepatocellular carcinoma. *Oncogene*. Sep 18 2008;27(42):5578-89. doi:10.1038/onc.2008.168

7. Mínguez B, Hoshida Y, Villanueva A, et al. Gene-expression signature of vascular invasion in hepatocellular carcinoma. *Journal of hepatology*. Dec 2011;55(6):1325-31. doi:10.1016/j.jhep.2011.02.034

8. Yan X, Chen M, Xiao C, et al. Effect of unfolded protein response on the immune infiltration and prognosis of transitional cell bladder cancer. *Annals of medicine*. Dec 2021;53(1):1048-1058. doi:10.1080/07853890.2021.1918346

9. Ritchie ME, Phipson B, Wu D, et al. limma powers differential expression analyses for RNA-sequencing and microarray studies. *Nucleic acids research*. Apr 20 2015;43(7):e47. doi:10.1093/nar/gkv007

10. Morris TJ, Butcher LM, Feber A, et al. ChAMP: 450k Chip Analysis Methylation Pipeline. *Bioinformatics (Oxford, England)*. Feb 1 2014;30(3):428-30. doi:10.1093/bioinformatics/btt684

11. Wu T, Hu E, Xu S, et al. clusterProfiler 4.0: A universal enrichment tool for interpreting omics data. *Innovation (Cambridge (Mass))*. Aug 28 2021;2(3):100141. doi:10.1016/j.xinn.2021.100141
