# Supplementary figures and images for: DNMT3L inhibits hepatocellular carcinoma progression through DNA methylation of CDO1: insights from big data to basic research
Source: J Transl Med. 2024 Feb 2;22:128. doi: 10.1186/s12967-024-04939-9 (PMC10837993; doi:10.1186/s12967-024-04939-9)

Figure S1

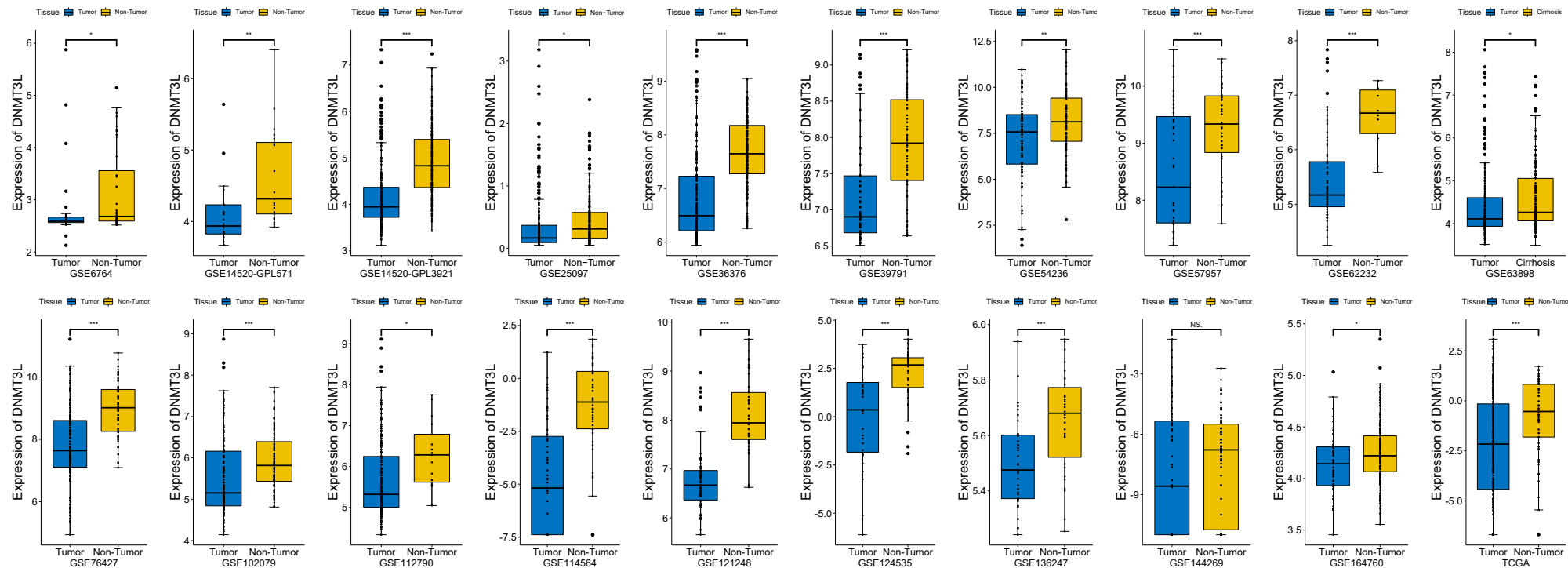

Supplement: Supplementary file 2 — Additional file 2: Figure S1. DNMT3L expression in HCC and non-tumor tissues in 20 datasets. *P < 0.05, **P < 0.01, ***P < 0.001. NS, no significant. [file 12967_2024_4939_MOESM2_ESM.pdf]

Figure S2

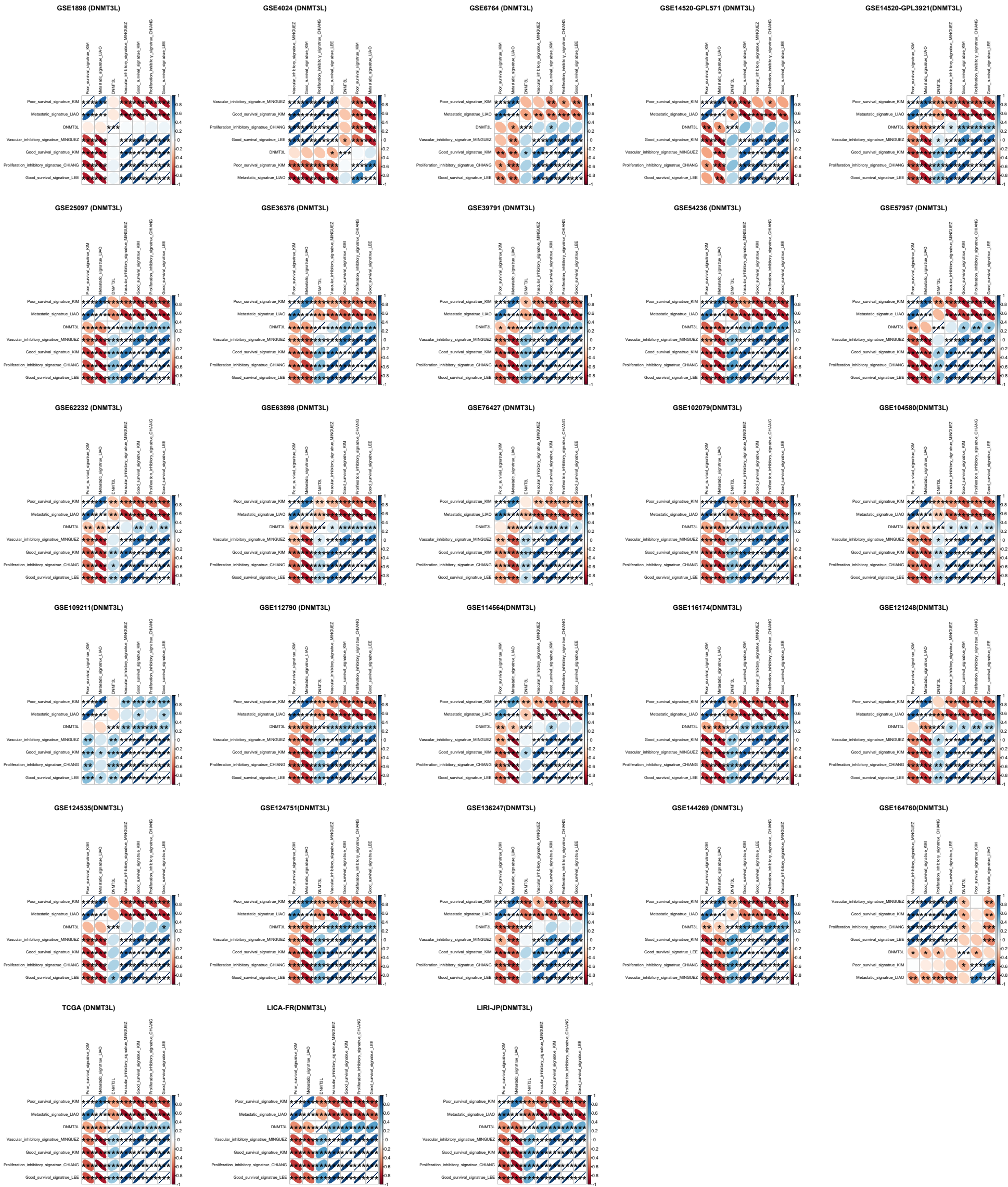

Supplement: Supplementary file 3 — Additional file 3: Figure S2. Correlations between DNMT3L expression and HCC signatures in 28 datasets. *P < 0.05, **P < 0.01, ***P < 0.001. [file 12967_2024_4939_MOESM3_ESM.pdf]

Figure S3

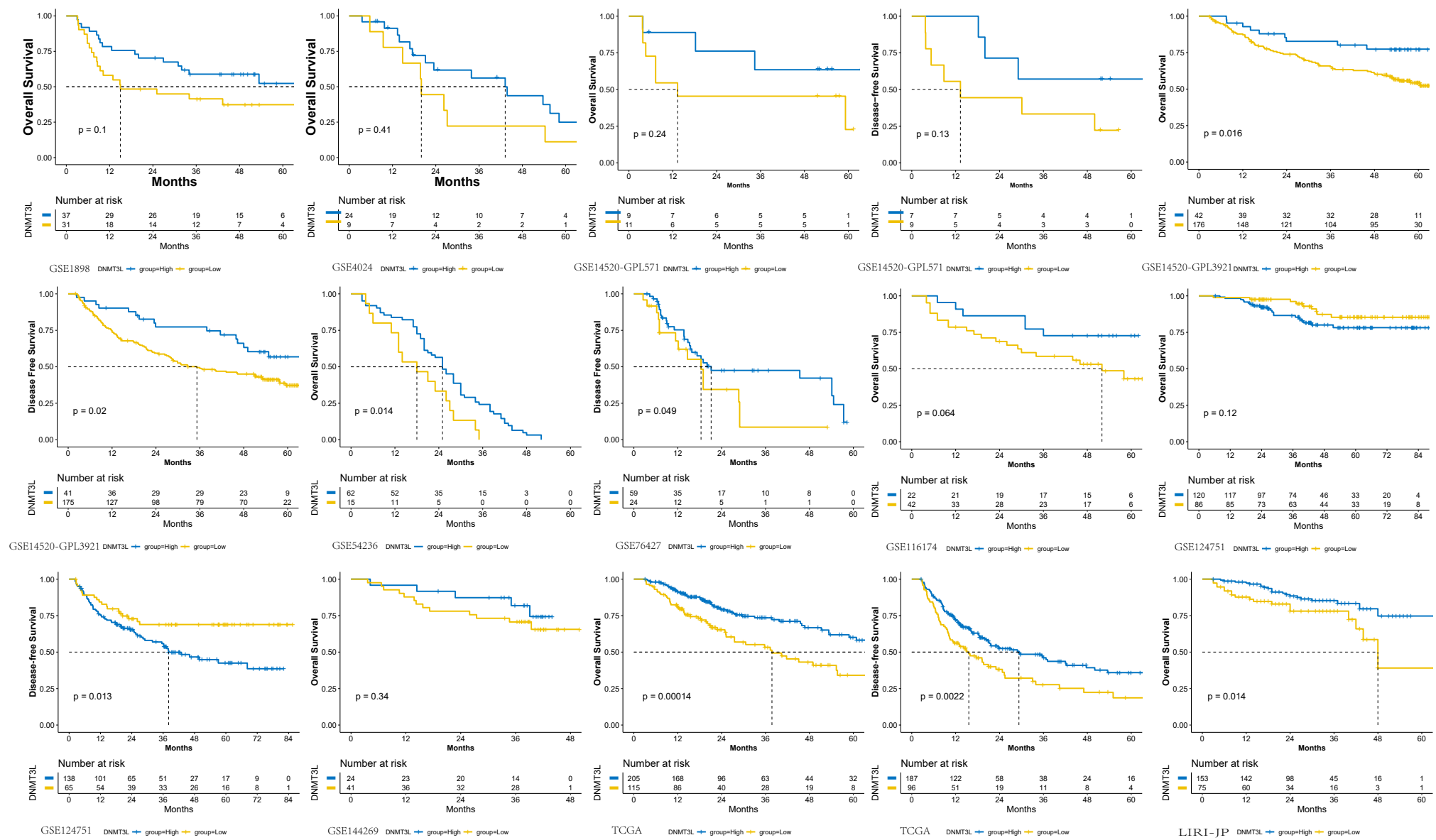

Supplement: Supplementary file 4 — Additional file 4: Figure S3. The relationship between DNMT3L expression and overall survival or disease-free survival in 11 datasets. [file 12967_2024_4939_MOESM4_ESM.pdf]

Figure S4

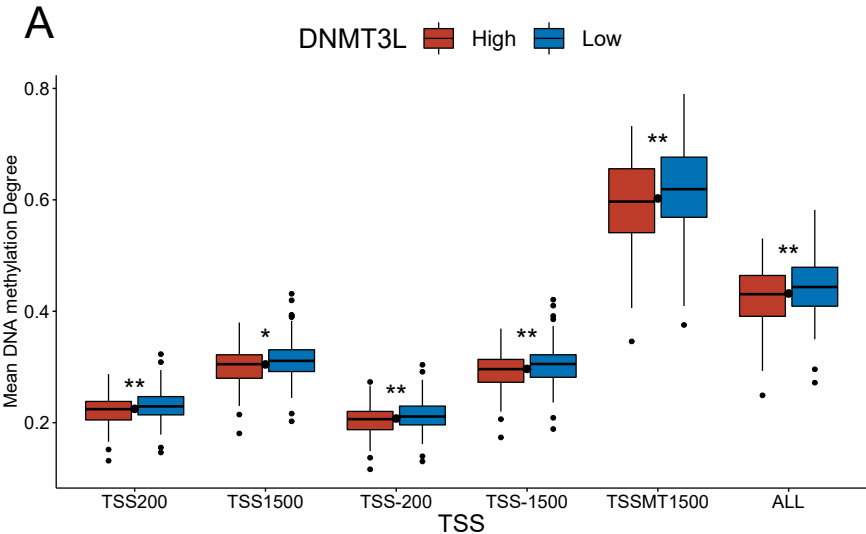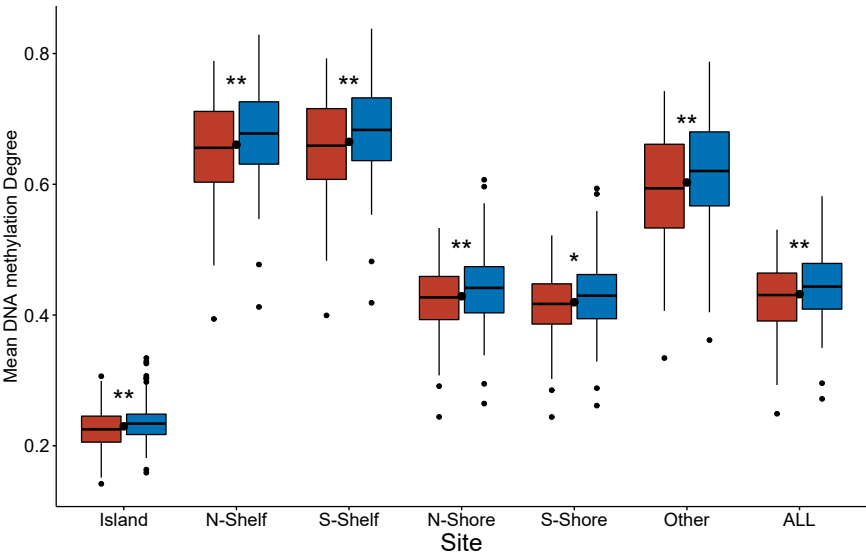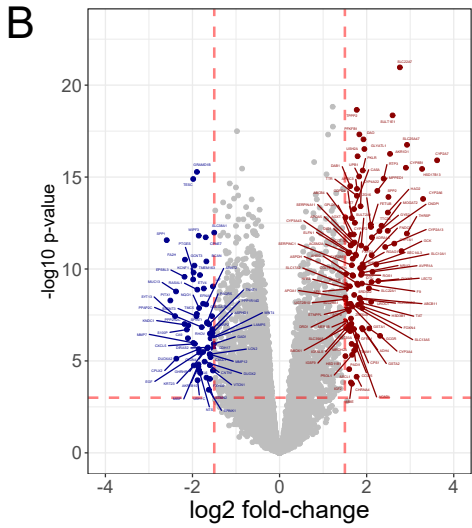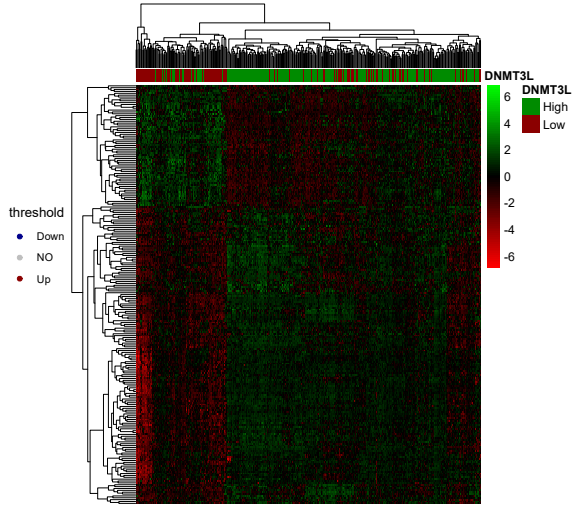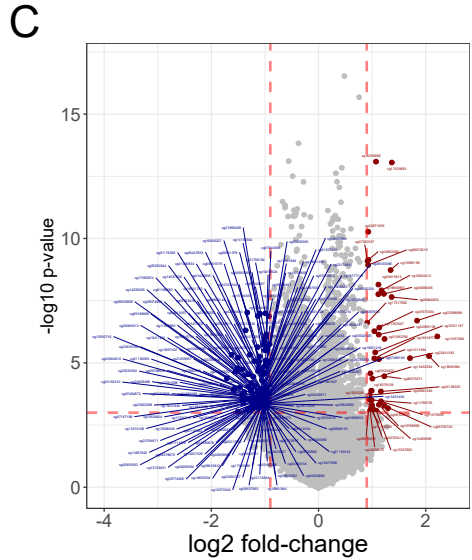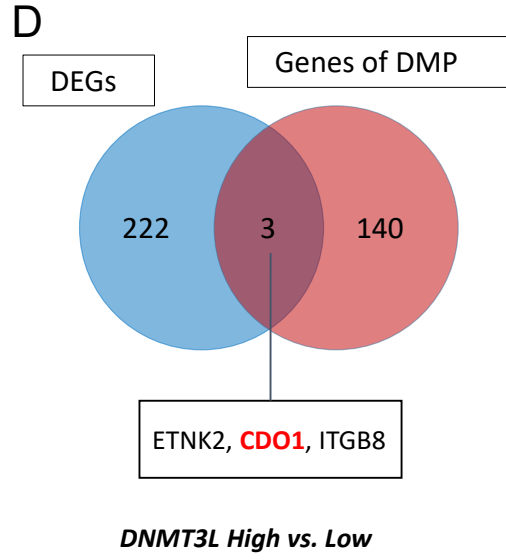

Supplement: Supplementary file 5 — Additional file 5: Figure S4. Screening of downstream target of DNMT3L. A, The relationship between DNMT3L expression and DNA methylation in TCGA. B, Volcano and heat-map plots show DEGs between DNMT3L-low and high groups in TCGA. C, Volcano plot show DMSs between DNMT3L-low and high groups in TCGA. D, Schematic of the selection for the downstream target of DNMT3L. TSS, transcription start site. TSS200: 0–200 bps downstream of TSS. TSS1500: 200–1500 bps downstream of TSS. TSS-200: 0–200 bps upstream of TSS. TSS-1500: 200–1500 bps upstream of TSS. TSSMT1500, more than 1500 bps from TSS. ALL, the whole DNA fragment. Island, CpG islands. N-Shelf, 2–4 kb upstream of CpG islands. S-Shelf, 2–4 kb downstream of CpG islands. N-Shore, 0–2 kb upstream of CpG islands. S-Shore, 0–2 kb downstream of CpG islands. Other: sites not on CpG islands, shelf and shore regions. DEGs, differential expressed genes. DMSs, differential methylation sites. *P < 0.05, **P < 0.01. [file 12967_2024_4939_MOESM5_ESM.pdf]

Figure S5

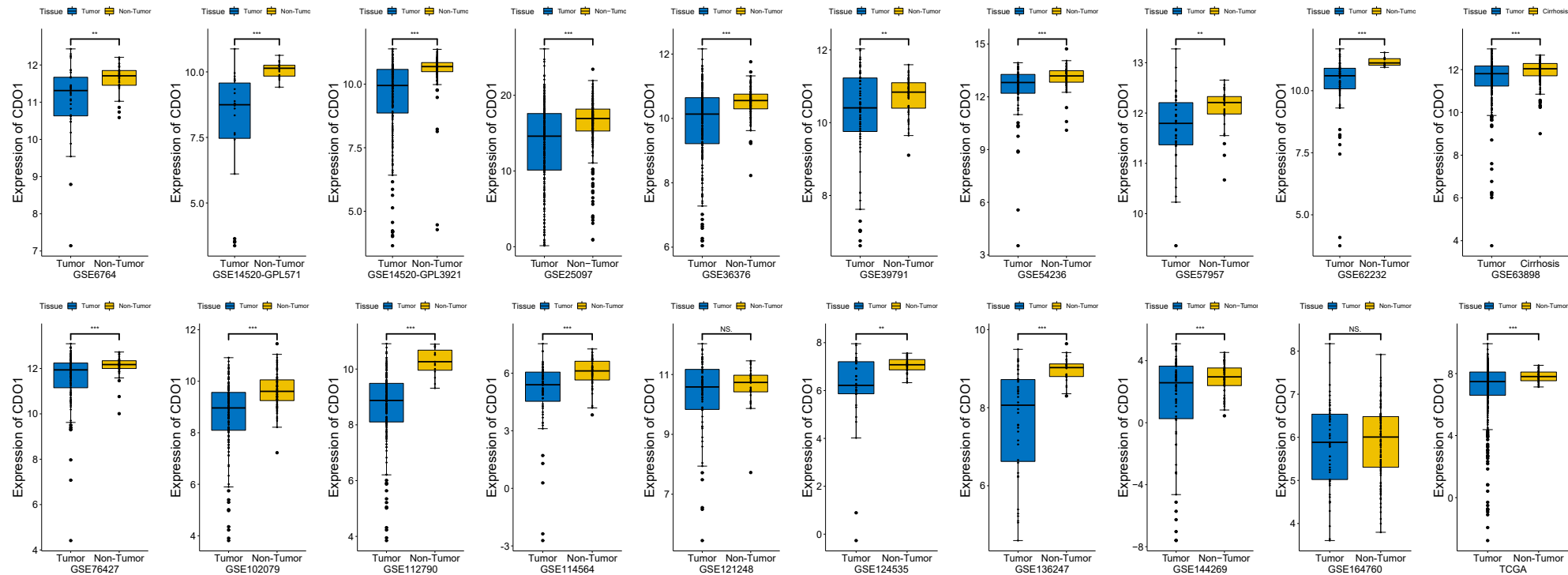

Supplement: Supplementary file 6 — Additional file 6: Figure S5. CDO1 expression in HCC and non-tumor tissues in 20 datasets. *P < 0.05, **P < 0.01, ***P < 0.001. NS, no significant. [file 12967_2024_4939_MOESM6_ESM.pdf]

Figure S6

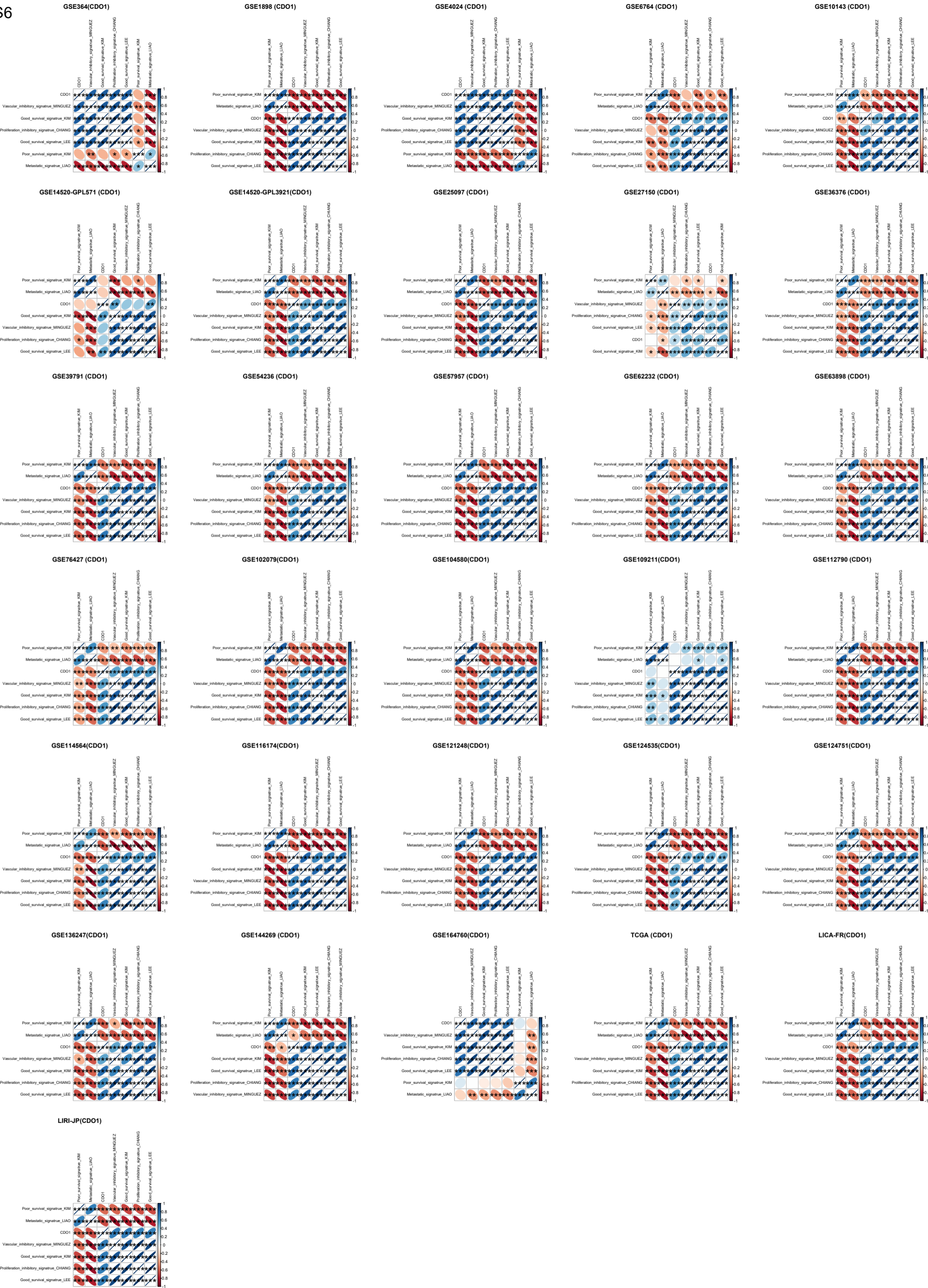

Supplement: Supplementary file 7 — Additional file 7: Figure S6. The relationship between CDO1 expression and HCC signatures in 31 datasets. *P < 0.05, **P < 0.01, ***P < 0.001. [file 12967_2024_4939_MOESM7_ESM.pdf]

Figure S7

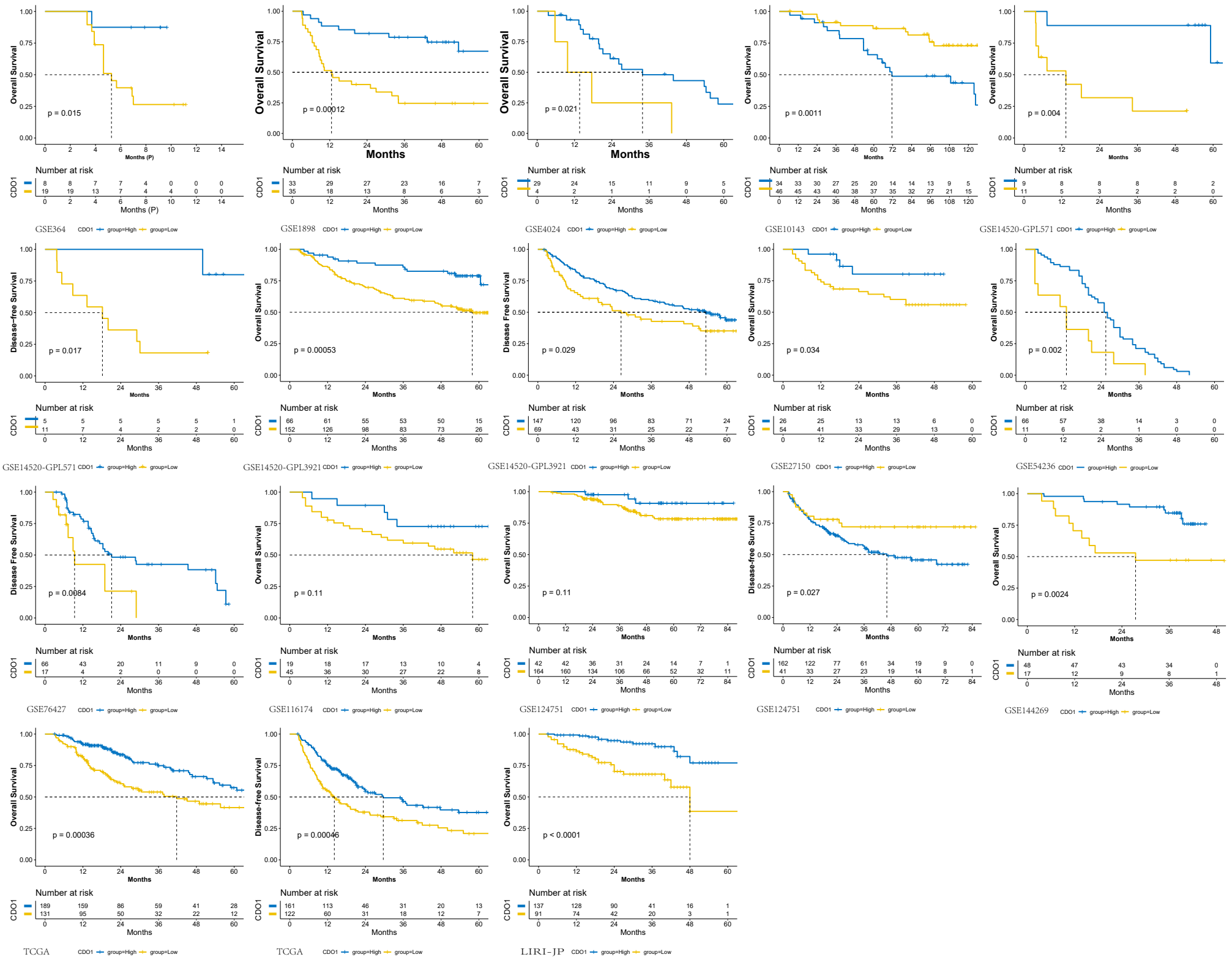

Supplement: Supplementary file 8 — Additional file 8: Figure S7. The relationship between CDO1 expression and overall survival or disease-free survival in 14 datasets. [file 12967_2024_4939_MOESM8_ESM.pdf]

Figure S9

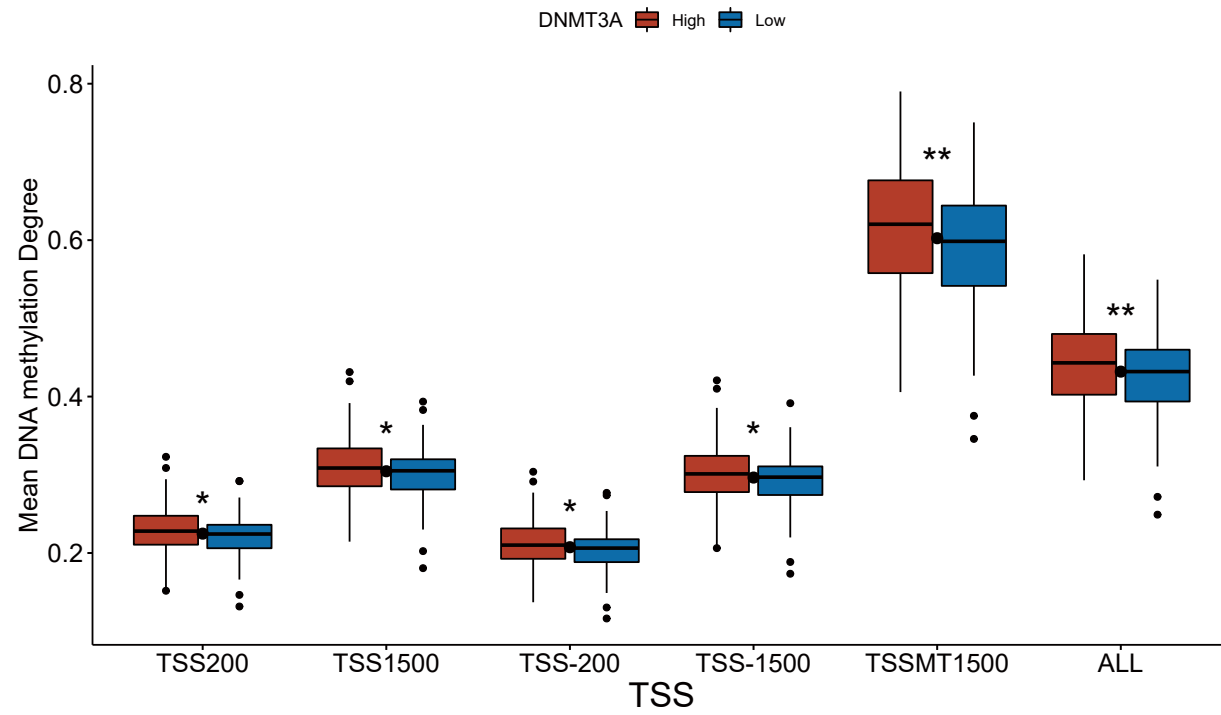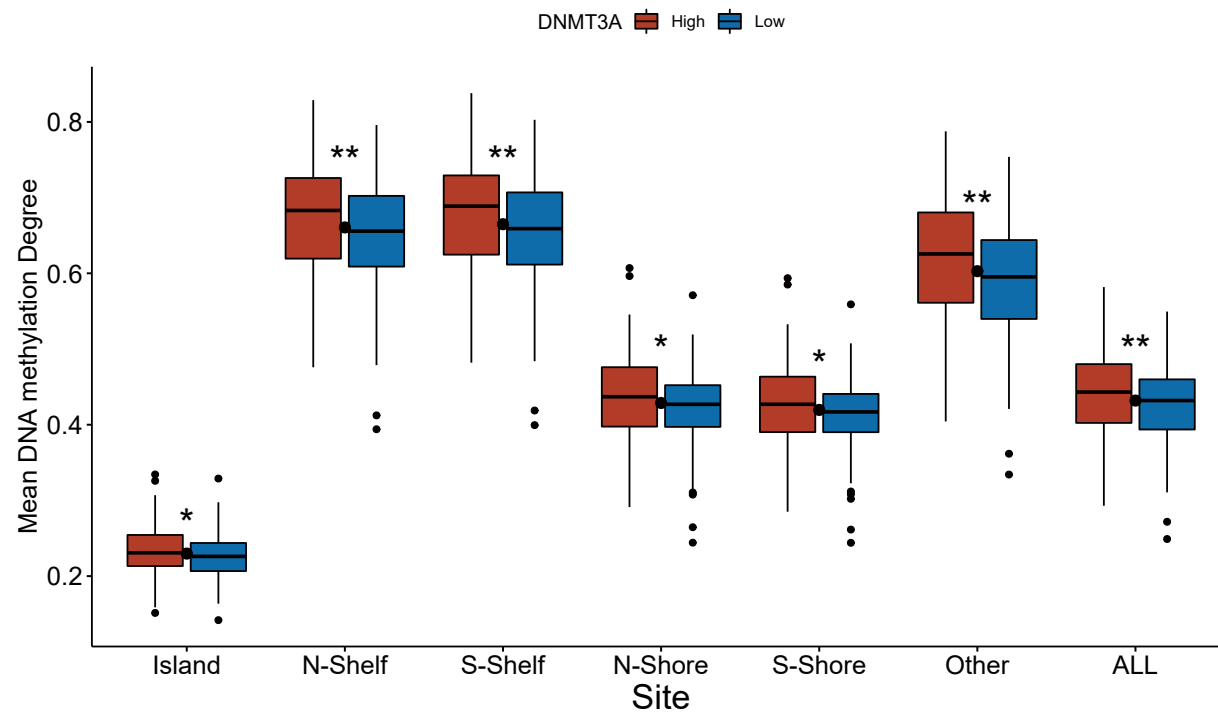

Supplement: Supplementary file 10 — Additional file 10: Figure S9. The relationship between DNMT3A expression and DNA methylation in TCGA. TSS, transcription start site. TSS200: 0–200 bps downstream of TSS. TSS1500: 200–1500 bps downstream of TSS. TSS-200: 0–200 bps upstream of TSS. TSS-1500: 200–1500 bps upstream of TSS. TSSMT1500, more than 1500 bps from TSS. ALL, the whole DNA fragment. Island, CpG islands. N-Shelf, 2–4 kb upstream of CpG islands. S-Shelf, 2–4 kb downstream of CpG islands. N-Shore, 0–2 kb upstream of CpG islands. S-Shore, 0–2 kb downstream of CpG islands. Other: sites not on CpG islands, shelf and shore regions. *P < 0.05, **P < 0.01. [file 12967_2024_4939_MOESM10_ESM.pdf]

Figure S10

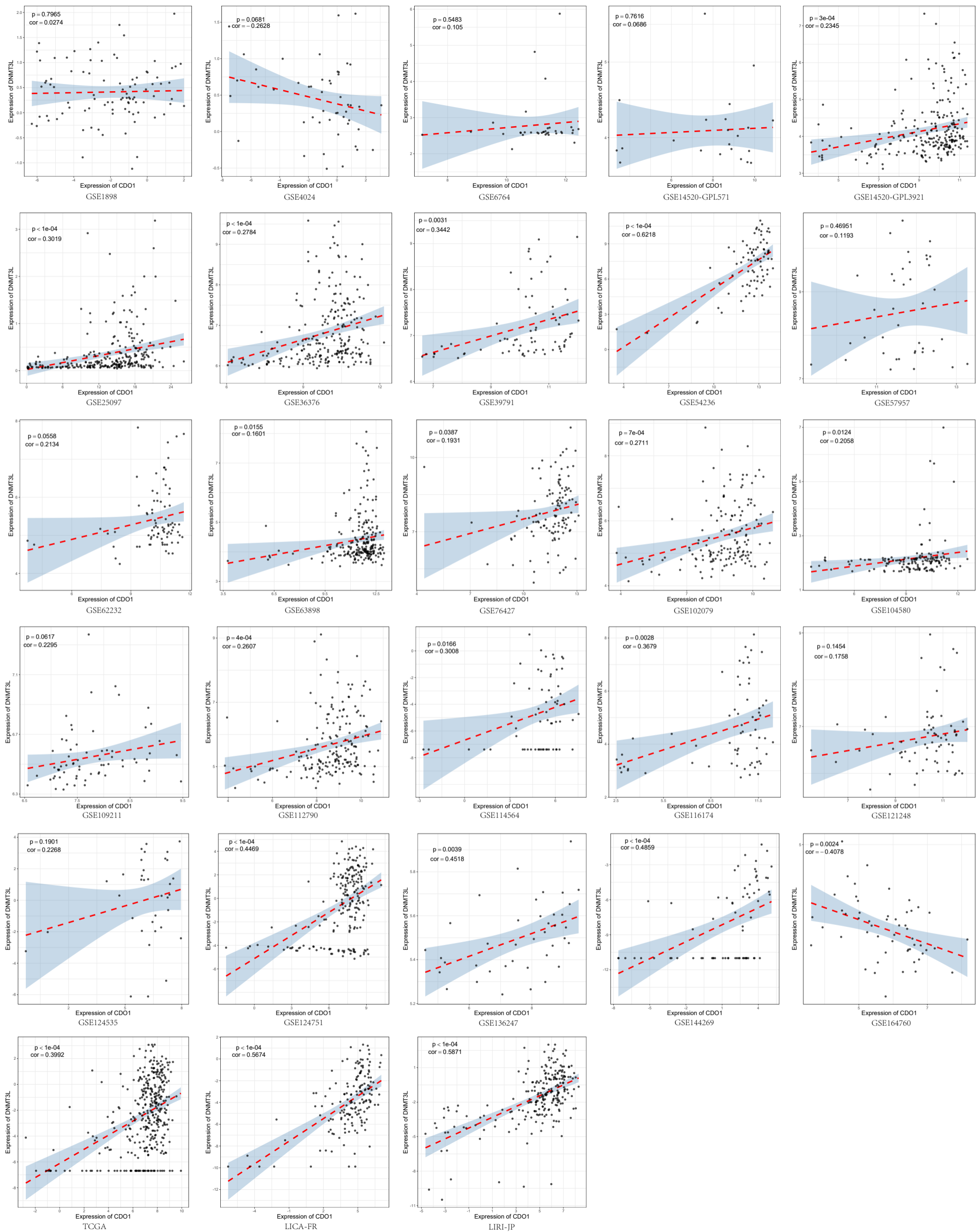

Supplement: Supplementary file 11 — Additional file 11: Figure S10. Correlations between DNMT3L expression and CDO1 expression in 28 datasets. [file 12967_2024_4939_MOESM11_ESM.pdf]

Figure S11

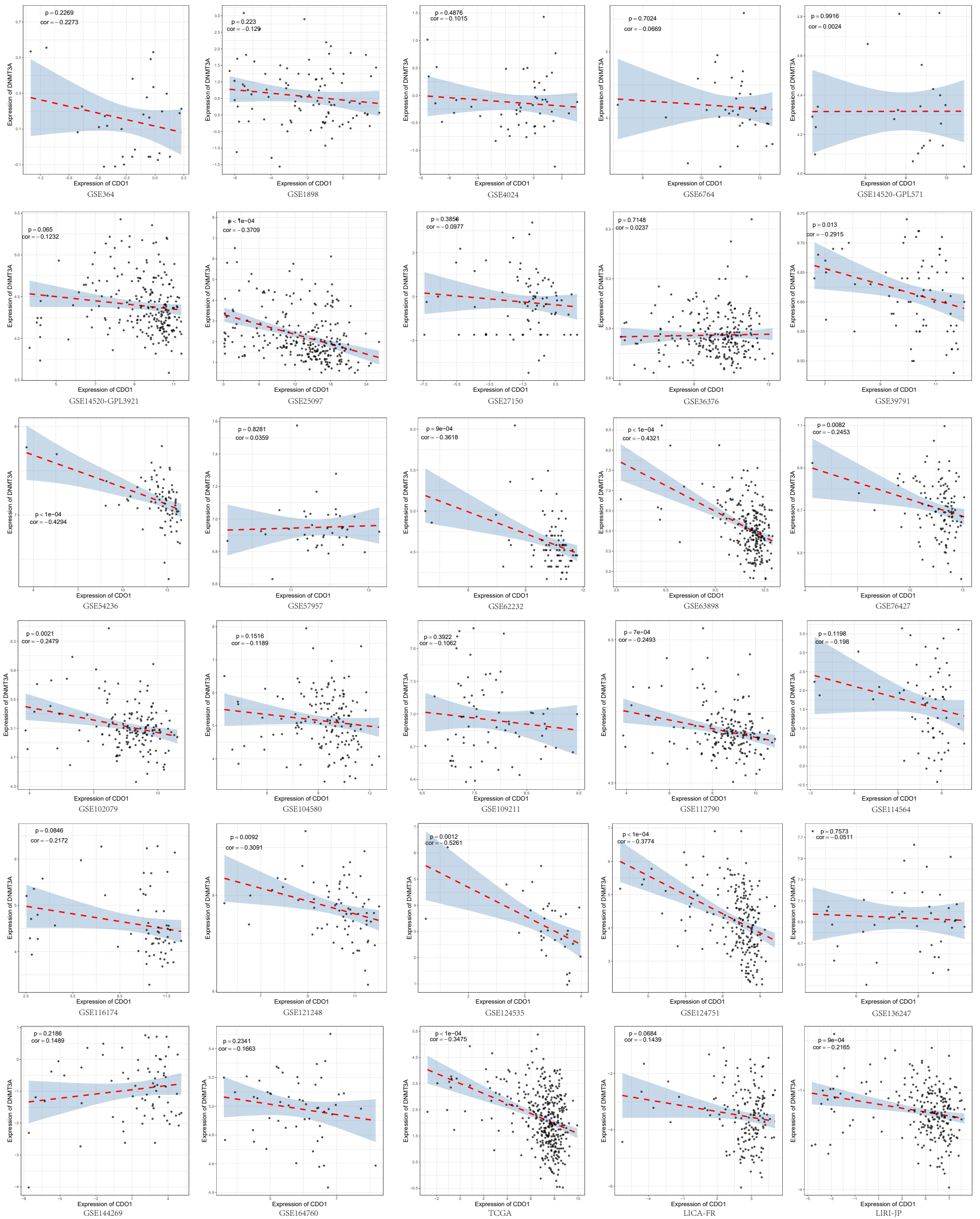

Supplement: Supplementary file 12 — Additional file 12: Figure S11. The relationship between DNMT3A expression and CDO1 expression in 30 datasets. [file 12967_2024_4939_MOESM12_ESM.pdf]

Figure S12

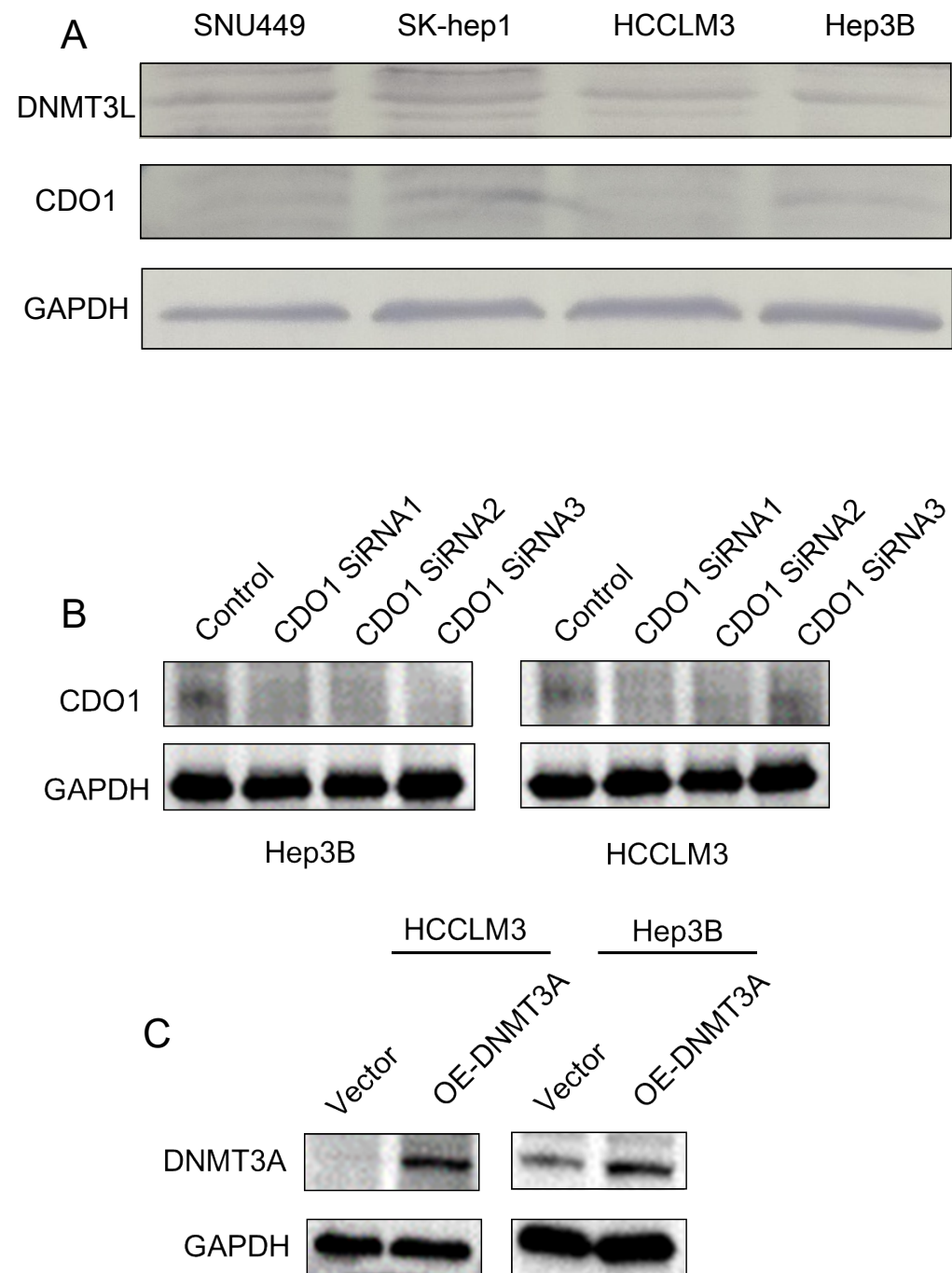

Supplement: Supplementary file 13 — Additional file 13: Figure S12. A, Western-blot analysis of DNMT3L and CDO1 expression in SNU449, SK-hep1, HCCLM3 and Hep3B cells. B, Western-blot analysis of CDO1 expression in HCCLM3 and Hep3B cells with transfected with CDO1 siRNA or the control. C, Plasmid transfection up-regulates DNMT3A expression in HCCLM3 and Hep3B cells. [file 12967_2024_4939_MOESM13_ESM.pdf]
