# Supplementary material for: DNMT3L inhibits hepatocellular carcinoma progression through DNA methylation of CDO1: insights from big data to basic research
Source: J Transl Med. 2024 Feb 2;22:128. doi: 10.1186/s12967-024-04939-9 (PMC10837993; doi:10.1186/s12967-024-04939-9)
Supplement: Supplementary file 9 — Additional file 9: Figure S8. GSVA, KEGG and GO analysis of CDO1. A, Relationship between CDO1 and hallmark gene sets. B, Correlation of CDO1 with bile acid metabolism, fatty acid metabolism, xenobiotic metabolism, and G2M checkpoint in TCGA. C, The top 200 genes with the highest correlation to CDO1. D, The top 10 KEGG enrichment-pathways associated with CDO1. E, The top 10 GO enrichment-pathways associated with CDO1. Cor, correlation coefficient. Negative, negative correlation. Positive, positive correlation. [file 12967_2024_4939_MOESM9_ESM.pdf]

Figure S8

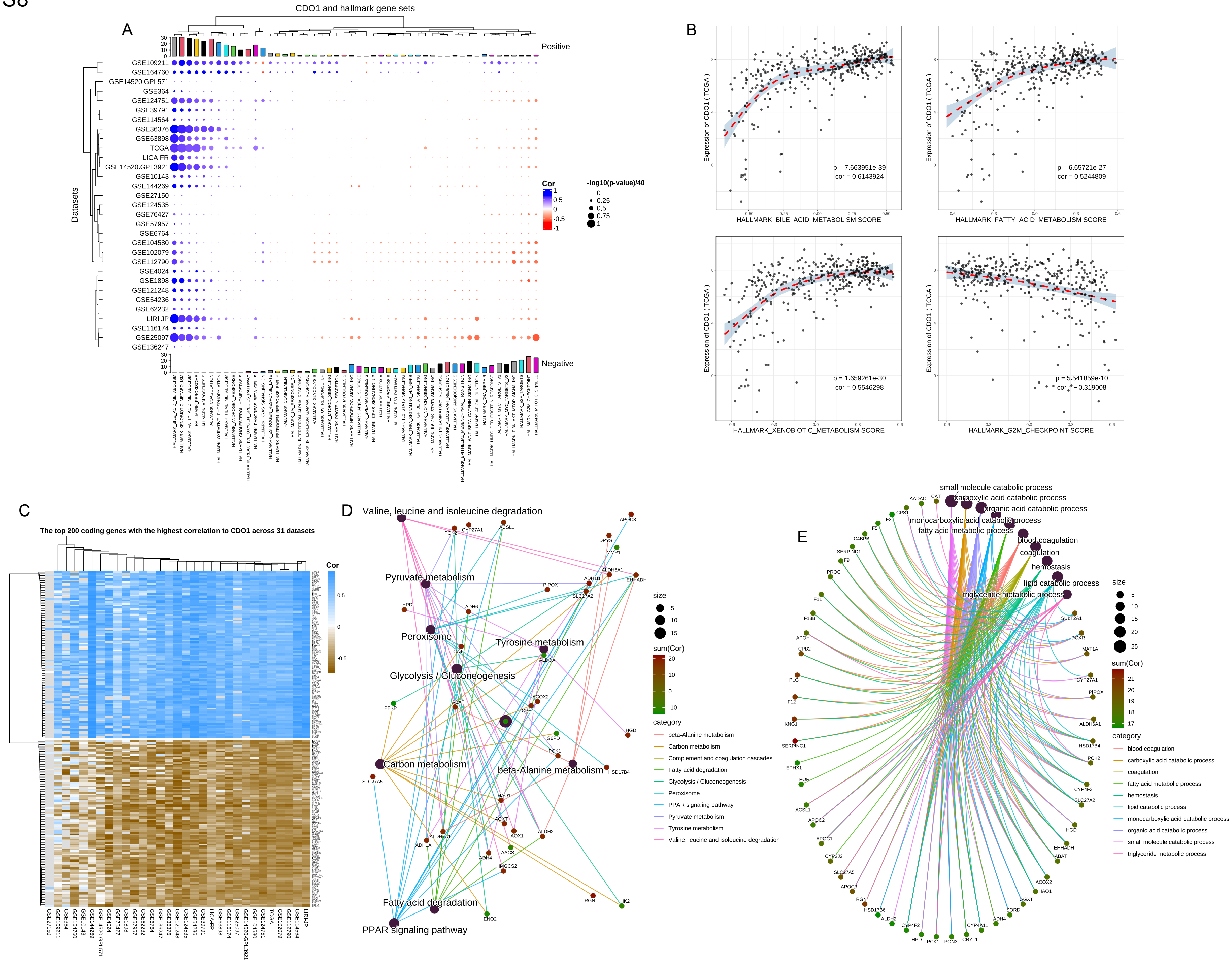

CDO1 and hallmark gene sets
A
Positive
Negative
Datasets
Cor
-log10(p-value)/40
B
p = 7.663951e-39
cor = 0.6143924
HALLMARK_BILE_ACID_METABOLISM SCORE
p = 6.65721e-27
cor = 0.5244809
HALLMARK_FATTY_ACID_METABOLISM SCORE
p = 1.659261e-30
cor = 0.5546298
HALLMARK_XENOBIOTIC_METABOLISM SCORE
p = 5.541859e-10
cor = −0.319008
HALLMARK_G2M_CHECKPOINT SCORE
Expression of CDO1 (TCGA)
C
The top 200 coding genes with the highest correlation to CDO1 across 31 datasets
Cor
D
Valine, leucine and isoleucine degradation
Pyruvate metabolism
Peroxisome
Tyrosine metabolism
Glycolysis / Gluconeogenesis
Carbon metabolism
beta-Alanine metabolism
Fatty acid degradation
PPAR signaling pathway
size
sum(Cor)
category
E
small molecule catabolic process
carboxylic acid catabolic process
organic acid catabolic process
monocarboxylic acid catabolic process
fatty acid metabolic process
blood coagulation
coagulation
hemostasis
lipid catabolic process
triglyceride metabolic process
size
sum(Cor)
category
